# Beyond the neuron: the role of glial cells in viral neuropathogenesis

**DOI:** 10.1128/jvi.01782-25

**Published:** 2026-06-22

**Authors:** Chloe R. Koon, Megan Culler Freeman

**Affiliations:** 1 Program in Microbiology & Immunology, University of Pittsburgh 6614, Pittsburgh, Pennsylvania, USA; 2 Department of Pediatrics, University of Pittsburgh School of Medicine 12317, Pittsburgh, Pennsylvania, USA; 3 UPMC Children’s Hospital of Pittsburgh 505599, Pittsburgh, Pennsylvania, USA; Indiana University Bloomington, Bloomington, Indiana, USA

**Keywords:** neurotropic, virus, organoid, astrocyte, microglia, oligodendrocyte, neural progenitor cell

## Abstract

The human central nervous system (CNS) is multicellular and complex. Sequelae of acute or chronic viral CNS infections can last a lifetime, and therapeutics are limited. Neurotropic viral infections can cause many manifestations, including encephalitis, meningitis, and paralysis. Most studies of neurotropic viruses focus on infection, dysfunction, and cellular death of neurons; however, neurons comprise only half of the cells of the human CNS. The remainder of the CNS is made of glial cells, such as astrocytes, microglia, oligodendrocytes, and neural progenitor cells (NPCs), which can give rise to neurons or glia. Glial cells serve critical maintenance and neuroprotective roles, including in response to infections. This review describes what is currently known about acute neurotropic viral infection of non-neuronal cells for human viral pathogens. Recent development of techniques to assess infection at the single-cell level, as well as increased complexity of human multicellular models of the brain and spinal cord, will expand our understanding of viral tropism and neuropathogenesis, potentially revealing novel therapeutic targets for these severe infections.

## INTRODUCTION

Viruses are a well-established cause of neurological conditions, including encephalitis, meningitis, paralytic myelitis, and others (1). These conditions occur due to infection of cells within the brain and spinal cord, which comprise the central nervous system (CNS). Viruses across multiple disparate families can gain access to the CNS and cause acute manifestations. Populations with a higher risk for severe neurological illness include young children, immunocompromised individuals, and older adults (2, 3). Infection during pregnancy can cause fetal neurological disease and impede neurodevelopment. Neurologic injury during viral infection is mediated by direct cytopathic effects in combination with pathogenesis from responding immune cells. While some viral infections have been associated with triggering immune-mediated neurologic disorders, such as multiple sclerosis and Guillain-Barré syndrome, this review will focus on acute neurological infections and their associated neuropathogenesis (1, 4–7).

The CNS is comprised of multiple specialized cell types organized as neurons and non-neuronal cells, predominantly glial cells. Neurons generate electrical signals to transmit information through the body. Neuron function is supported by glial cells. Glial cells include astrocytes, microglia, and oligodendrocytes. Astrocytes and microglia both regulate CNS homeostasis and immune responses, while oligodendrocytes create the myelin sheath to accelerate neuronal signal conduction (8). Neural progenitor cells (NPCs) are precursors for neurons and glial cells during development and beyond (9).

Most research on viral neuropathogenesis has focused on viral infection of neurons and subsequent alterations in function or viability, despite glial cells being as numerous as neurons in the human CNS (10–14). While most of the viruses included in this review infect neurons, they also infect glial cells and can result in dysfunction and death, likely contributing to neuropathogenesis. The role of glial cells in viral neuropathogenesis and how infection of these specialized cells contributes to disease is understudied, potentially due to challenges in model systems.

**Fig 1:**
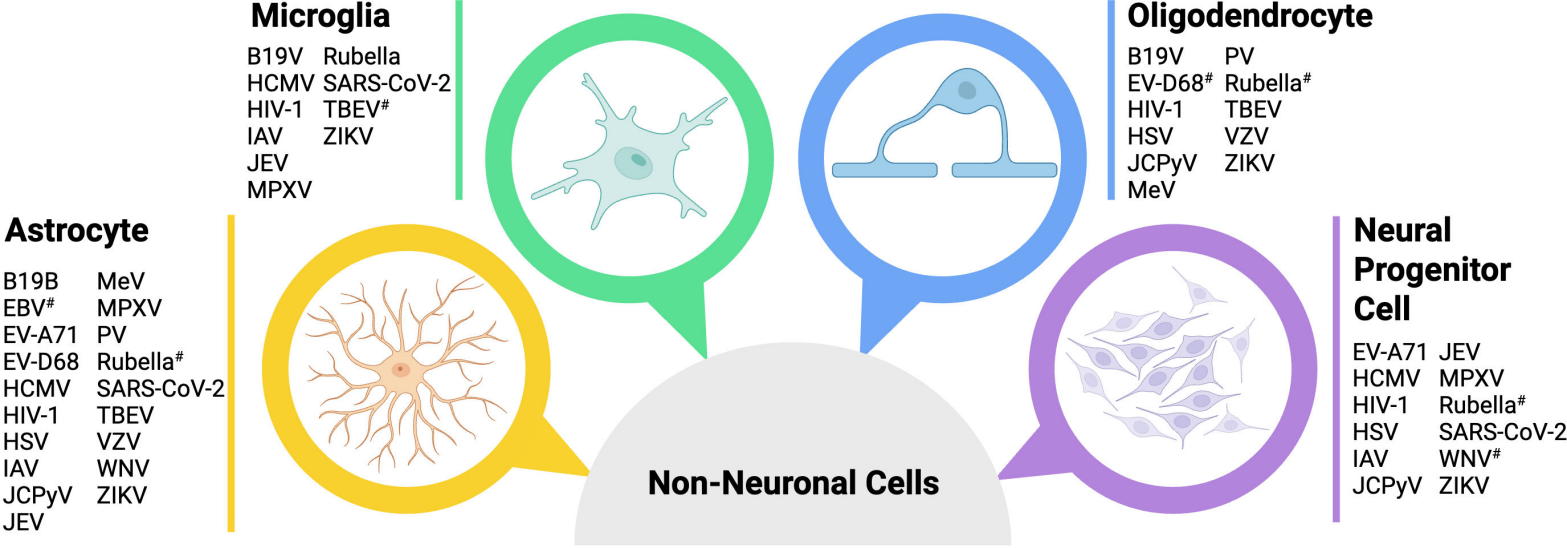
Neurotropic viruses that infect non-neuronal CNS cell types. Viruses discussed in the review that infect astrocytes, microglia, oligodendrocytes, and/or NPCs. # indicates that evidence for a particular virus productively infecting that cell type is limited. Created with BioRender.

Recent technological advances have led to new opportunities to discover the role of multiple cell types in viral pathogenesis. Single-cell RNA sequencing and transcriptomics have allowed further distinction of cell types within the CNS and how these cell types are modified by disease (15, 16). This level of transcriptomic detail has expanded cell subtypes beyond what was previously known. In combination with human stem cell–derived CNS cell lines and other multicellular human organoids of the brain and spinal cord, new work has identified additional complexity of neurotropic viral infections beyond the neuron (17–23). This review examines the current state of the literature as it relates to the role of glial cells in neuropathogenesis of human neurotropic viruses (Fig. 1, Table 1). We also present remaining gaps in knowledge and how research using state-of-the-art model systems can bridge these gaps to understand the role of these critical supporting cells in disease. A better understanding of the role of these underrecognized cell types during viral neuropathogenesis and underlying convergent viral mechanisms could lead to new therapeutic targets for neurotropic viral infections.

**TABLE 1:** Neurotropic viruses that infect non-neuronal CNS cell types^*a*^

Family	Virus	Astrocytes	Microglia	Oligodendrocyte	NPCs	Neurons
(+) Sense RNA						
*Coronaviridae*	Severe acute respiratory syndrome coronavirus 2 (SARS-CoV-2)	**Yes** (24–31)	**Yes** (32)	–	**Yes** (33, 34)	**Yes**
*Flaviviridae*	Japanese encephalitis virus (JEV)	**Yes** (35–37)	**Yes** (38–41)	–	**Yes** (42–44)	**Yes**
	Tick-borne encephalitis virus (TBEV)	**Yes** (45–47)	Limited (48)	**Yes** (45)	–	**Yes**
	West Nile virus (WNV)	**Yes** (49–55)	–	**–**	Limited (56)	**Yes**
	Zika virus (ZIKV)	**Yes** (4, 57–66)	**Yes** (23, 67, 68)	**Yes** (69)	**Yes** (70–78)	**Yes**
*Matonaviridae*	Rubella virus	Limited (79–81)	**Yes** (82)	Limited (83)	Limited (84)	–
*Picornaviridae*	Enterovirus A71 (EV-A71)	**Yes** (85–87)	–	–	**Yes** (88, 89)	**Yes**
	Enterovirus D68 (EV-D68)	**Yes** (90–94)	–	Limited (94)	–	**Yes**
	Poliovirus (PV)	**Yes** (95)	–	**Yes** (95)	–	**Yes**
(-) Sense RNA						
*Orthomyxoviridae*	Influenza virus (IAV)	**Yes** (96–101)	**Yes** (98, 102)	–	**Yes** (20)	**Yes**
*Paramyxoviridae*	Measles virus (MeV)	**Yes** (103–106)	–	**Yes** (107)	–	**Yes**
Retrovirus						
*Retroviridae*	Human immunodeficiency virus 1 (HIV-1)	**Yes** (108–115)	**Yes** (116–120)	**Yes** (121, 122)	**Yes** (123–125)	Limited
DNA						
*Herpesviridae*	Epstein-Barr virus (EBV)	Limited (126)	–	–	–	**Yes**
	Herpes simplex virus 1/2 (HSV)	**Yes** (127–130)	**–**	**Yes** (131–134)	**Yes** (135–137)	**Yes**
	Human cytomegalovirus (HCMV)	**Yes** (138–140)	**Yes** (138, 140, 141)	–	**Yes** (22, 142–147)	**Yes**
	Varicella-zoster virus (VZV)	**Yes** (148–151)	–	**Yes** (152)	–	**Yes**
*Parvoviridae*	Parvovirus B19 (B19V)	**Yes** (153, 154)	**Yes** (154, 155)	**Yes** (154)	–	**Yes**
*Polyomaviridae*	JC polyomavirus (JCPyV)	**Yes** (156–161)	–	**Yes** (158, 162–166)	**Yes** (157, 167, 168)	**Yes**
*Poxviridae*	Mpox virus (MPXV)	**Yes** (21, 169, 170)	**Yes** (170)	–	**Yes** (21, 169)	**Yes**

^*a*^ Viruses discussed in the review that infect astrocytes, microglia, oligodendrocytes, and/or NPCs. Boldface denotes that there is evidence for that virus infecting that cell type. –, no evidence to support that virus infecting that cell type.

## ASTROCYTES

Astrocytes are one of the most abundant cell types within the CNS and are the largest population of glial cells (171). In the late 1800s, it was thought that astrocytes solely caused pathology; however, within the past few decades, this paradigm shifted when it was discovered that astrocytes regulate daily CNS homeostasis (172). This includes forming the blood–brain barrier and regulating metabolites and neurotransmitters to ensure proper neuronal function (172, 173). As part of the blood–brain barrier, astrocytes are one of the first CNS cell types to encounter a pathogen. Astrocytes are innate immune cells that recognize and respond to infection by producing cytokines and chemokines to recruit the adaptive immune response, activate resident microglia, and change blood flow. A subtype of reactive astrocytes proliferates to form protective barriers around damaged areas in the CNS to sequester infection and inflammation (174). Loss of this protective astrocytic barrier in a spinal cord injury model results in increased spread of inflammation throughout the spinal cord, increased neuron loss, and diminished functional recovery (175). The impact of border-forming astrocytes has not been evaluated in infection models (176).

Various astrocyte models exist, including neural tissues, cell lines, primary astrocytes, induced pluripotent stem cell (iPSC)-derived astrocytes, organoid models, and animal models. Each of these models has advantages and disadvantages. While potentially the most relevant, human neural tissues are rare and usually acquired at autopsy; therefore, they are only representative of the latest stage of disease. Cell lines are often of nonhuman origin, so utility is limited based on the host range of a particular virus (177, 178). Primary astrocytes retain their characteristics for a limited number of passages, can be difficult to culture, and may vary based on source. Human iPSC-derived astrocytes are a reliable model that can retain characteristics of their patient of origin, but can be challenging to differentiate and take time to establish (179, 180). Primary astrocytes and other monocultures allow for the study of direct effects of viral infection on this cell type, but lack the complexity of multicellular models (181). Multicellular human models, such as human CNS organoids, can convey the relationship between CNS cell types but are relatively nascent in development, are organized differently than native tissue, and do not include components of the adaptive immune system (182). Animal models allow for the study of viral dissemination and for contributions of the immune system; however, not all human viruses readily infect animals without modification.

### RNA viruses

Multiple viruses in the *Flaviviridae* family have astrocyte tropism, including West Nile virus (WNV), Zika virus (ZIKV), tick-borne encephalitis virus (TBEV), and Japanese encephalitis virus (JEV) (49). There is evidence of flaviviruses inducing astrocyte cell death, altered cellular function, and cytokine and chemokine production. Neural tissues of deceased individuals with WNV encephalomyelitis had detectable WNV capsid in both neurons and astrocytes. Rather than glial cell death, astrocytes secreted factors that induced apoptosis of nearby neurons (50). Another study found that in a sample from a patient with acute flaccid myelitis (AFM), WNV-infected astrocytes had decreased glutamate transporter expression, leading to less glutamate uptake by astrocytes and excessive extracellular glutamate, which caused neuron death via excitotoxicity (51). In an *ex vivo* spinal cord model, reactive astrocytes infected with WNV died via apoptosis (52). WNV-infected astrocytes produced chemokines, including CCL5 (C-C motif chemokine ligand 5), a pro-inflammatory chemokine, which was found at higher levels when infected with a highly pathogenic WNV, demonstrating that WNV astrocyte infectivity and activation may be strain specific (53–55).

ZIKV has been associated with microcephaly and other brain anomalies during congenital infection (6, 183–188). While the complete mechanism of neuropathogenesis for ZIKV is not known, astrocytes are infected across multiple human *in vitro* infection models and are thought to be a reservoir for ZIKV in the developing brain (57–60). Astrocytes become reactive during infection with disrupted functionality, including mitochondrial failure and gene expression changes that affect homeostatic regulation (4, 58, 59). Astrocytes may be preferentially infected by ZIKV, resulting in cell death; however, different strains of ZIKV have varying levels of infectivity of astrocytes and produce different antiviral responses (61–64). Mechanistically, ZIKV entry into astrocytes is promoted by AXL receptor tyrosine kinase (AXL), which initiates the immune response, with toll-like receptor 3 (TLR3) further promoting ZIKV infection and associated immune response (65, 66).

TBEV and JEV infect astrocytes *in vitro* (35, 45, 189). TBEV causes more neuron cell death than astrocyte cell death, but both cell types produce chemokines and cytokines upon infection (45, 46). Astrocyte-released cytokines appear to have a self-protective effect during TBEV infection, perhaps demonstrating a physiologic role rather than pathologic (47). JEV induces differential astrocytic gene expression as well as chemokine and cytokine production, including chemokine CCL5 (35–37). This suggests that CCL5 production may be conserved among flavivirus infection and contribute to strong pro-inflammatory signaling that can lead to damaging neuroinflammation. There are also indications that there could be strain-specific virulence within individual flavivirus species, such as WNV and ZIKV, in which stronger cellular responses are seen with specific strains (54, 55, 63, 64).

Viruses within the *Picornaviridae* family have been associated with astrocyte tropism and pathogenesis, including cell death and cytokine production (190). While poliovirus neuropathogenesis is primarily driven by neuron infection and death, poliovirus can infect human astrocytes, leading to apoptosis (95). Enterovirus D68 (EV-D68), a cause of AFM, has evidence of astrocyte infection in humans and *in vitro* (90). EV-D68-infected non-neuronal cells were identified in spinal cord autopsy tissue of a child who died of AFM, and EV-D68 infects primary human astrocyte cultures and human spinal cord organoids (91–94). Another neurotropic picornavirus, enterovirus A71 (EV-A71), preferentially infects astrocytes and causes the production of cytokines and excitatory neurotransmitters that contribute to neuropathogenesis by overstimulating neurons, creating excessive neuronal signaling (85). EV-A71 infection is most severe in infants and, concordant with this, infection of neonatal mice leads to greater cytokine and neurotransmitter production, leading to decreased survival (86). As for mechanisms of pathogenesis, the C5a-C5aR1 complement pathway in astrocytes drives neutrophil recruitment during EV-A71 infection, contributing to neuroinflammation (87).

Severe acute respiratory syndrome coronavirus 2 (SARS-CoV-2) has astrocyte tropism in human *in vitro* and mouse models, and infection significantly changes gene expression (24–26). There have been differences in gene expression regulation in astrocytes between variants as well (27). Most work has shown that SARS-CoV-2-infected astrocytes are a major source of neuroinflammation that can lead to both astrocytic and neuronal cell death (28–31).

Influenza virus (IAV) is a rare cause of neuropathology, and astrocyte infection is strain-specific (96, 97). Cytokine production from infected astrocytes occurs across strains, contributing to a proinflammatory immune response in both human and murine astrocytes *in vitro,* often associated with astrocyte cell death, though the degree of cytokine production varies (98–101). There is evidence of IAV-inducing apoptosis of infected astrocytes (98); however, in other studies, viral replication is found without production of infectious progeny (99, 100). IAV strain-specific variation could be associated with varying neuropathology and may be driven by differing infectivity of astrocytes.

Measles virus (MeV) can cause multiple types of acute or delayed encephalitis, including acute post-infectious measles encephalitis (APME), subacute sclerosing panencephalitis (SSPE), and measles inclusion-body encephalitis (MIBE) (191). MeV antigen has been identified in astrocytes of patients with SSPE (103, 104). MeV infects astrocytes *in vitro*, resulting in both acute and chronic infections, with upregulation or disruption of glial fibrillary acidic protein (GFAP), an astrocytic marker indicating activation (105, 106). MeV infection of astrocytes results in changes in the cellular cytoskeletal and inflammatory state of astrocytes that may have a long-lasting impact on the host.

Rubella and HIV-1 have evidence of astrocyte infection, but mechanistic studies are limited. Rubella infects astrocytes *in vitro*, but additional mechanistic detail is limited (79–81). HIV-1 can lead to multiple acute neurologic conditions, including meningitis, as well as ultimately encephalopathy (192, 193). Evidence from patient samples, as well as *in vitro* studies, suggests that HIV-1 can infect astrocytes (108–112). One study showed that infected astrocytes remained intact while uninfected bystander cells underwent apoptosis mediated through gap junctions between the astrocytes and other cells (113). Astrocytes may be acting as long-term HIV-1 reservoirs within the CNS (114, 115).

### DNA viruses

While parvovirus B19 is usually acquired in childhood, infected astrocytes have been identified in post-mortem tissue of elderly people with or without encephalopathy. This suggests long-term viral persistence in astrocytes; however, the significance of this is unclear (153, 154). Potential viral persistence in astrocytes, as highlighted by parvovirus B19 and HIV-1, demonstrates a role for astrocyte-specific viral evasion strategies of host detection, which require further evaluation.

Herpes simplex virus 1 and 2 (HSV-1/2), human cytomegalovirus (HCMV), varicella-zoster virus (VZV), and Epstein-Barr virus (EBV) are all viruses in the *Herpesviridae* family commonly associated with neurological manifestations, and each has astrocyte tropism (194). During acute, lytic replication, HSV-1/2 both infect astrocytes and cause programmed cell death in human brain slice cultures, but the specific pathway has not been characterized (127). HSV-2 infection of primary murine astrocytes caused mitochondrial dysfunction and cytoskeleton remodeling (128). HSV-1-infected astrocytes upregulate certain receptors for immune sensing and can produce chemokines and cytokines, including CXCL1 in a mouse model (C-X-C motif chemokine ligand 1), a factor involved in recruitment of immune cells through the blood–brain barrier (129, 130).

HCMV, the most common congenital infection, can cause hearing loss and developmental concerns related to viral neurotropism *in utero* (195). HCMV infects astrocytes *in vitro,* potentially preferentially, resulting in cell death via apoptosis (138, 139). HCMV-infected astrocytes produced chemokines, such as MCP-1 (monocyte chemoattractant protein-1) and IL-8 (interleukin-8), known to recruit immune cells (140). VZV readily infects cultured human astrocytes, though there may be permissibility differences between astrocytes from different CNS locations (148, 149). VZV-infected astrocytes have been identified in patients with encephalitis, with one case describing infected astrocytes that created a distinct border surrounding other infected cells (150, 151). Lastly, EBV infects different astrocyte cell lines (126). Clinically, there is some evidence that EBV-infected astrocytes may lead to the development of multiple sclerosis, though this is difficult to confirm given the ubiquity of EBV infection (5). Together, herpesviruses readily infect astrocytes, leading to astrocyte cell death, altered functionality, and cytokine production.

Mpox virus (MPXV) can cause neurological conditions, including encephalitis (196). MPXV infects human astrocytes *in vitro* and somewhat uniquely lacked productive infection in neurons in one study; however, another group discovered that MPXV infected both neurons and astrocytes in a neural organoid model (21, 169). As both studies used a viral isolate from the same clade, it is likely that the infection differences are related to the model systems investigated. MPXV-infected astrocytes succumbed to cell death through pyroptosis *in vitro* (170). Human polyomavirus 2, commonly referred to as John Cunningham (JC) polyomavirus (JCPyV), causes meningitis and encephalopathy but is best known for causing progressive multifocal leukoencephalopathy (PML), a severe demyelinating disease (197). Data from patient reports, as well as *in vitro* and *in vivo* studies, demonstrate that JCPyV infects astrocytes (156–158). JCPyV induces alterations in gene expression and protein levels that drive astrocyte dysregulation of cellular proliferation, signaling pathways, and inflammation (159, 160). One study found that the MAP/ERK pathway and protein DUSP1, involved in extracellular signal transduction, were needed for successful JCPyV infection of primary astrocytes, though this was not necessary for infection of an immortalized cell model (161). Work with MPXV and JCPyV demonstrates the importance of using human model systems with adequate complexity to recapitulate native cellular function.

Together, evidence from RNA and DNA viruses suggests that astrocytes are a primary target for viral replication in the CNS with associated cellular responses to infection. Astrocyte infection leads to astrocyte death, altered function or structure, and the production of cytokines and chemokines, which alter CNS homeostasis. Astrocytes also act as potential immune-protected reservoirs for infection with long-lasting consequences on human health.

## MICROGLIA

Microglia are CNS resident macrophages and a part of the innate immune system. Microglia support CNS development and neuron homeostasis through phagocytosis of dead cells or debris to maintain a healthy environment (198). Beyond maintenance, activated microglia are an important part of the neuroinflammatory response during CNS infection. Reactive microglia proliferate, phagocytose microbes and debris, and produce pro-inflammatory cytokines (199). While microglia and astrocytes have similar roles in the daily maintenance of the CNS, activated microglia aid in clearing CNS infection by initiating an adaptive immune response. This review will focus on viruses that infect microglia, as opposed to viruses that indirectly trigger microglial activation. Because microglia can phagocytose materials, viral detection within microglia has the potential caveat of being representative of phagocytosed virus rather than active infection.

Existing models for microglia include human neural tissues, cell lines, primary microglia, murine, and nonhuman primate models. Several immortalized microglial cell lines exist of human or murine origin that have been used to study viral infection, but they can vary in their level of response and functionality (200). While infected monocultures allow for specific examination of viral replication and infection of microglia themselves, in multicellular models, it is more difficult to determine whether microglia are directly infected or responding to infection of neighboring cells. Because CNS organoids are derived from the ectodermal lineage, microglia, which arise from mesoderm, do not develop. Microglia have been successfully co-cultured with CNS organoids for the study of viral pathogenesis in a more complex human model system (201, 202).

### RNA viruses

Viral infection of microglia has not been as thoroughly investigated as astrocytes, perhaps because of difficulties distinguishing their physiological response as an innate immune cell from their pathological response as an infected cell. As microglia act as the macrophages of the CNS, infection of these cells often leads to increased and sustained cytokine production. This occurs for ZIKA and TBEV, where infected microglia *in vitro* produce various cytokines, including IL-6 and MCP-1 (48, 67, 68). SARS-CoV-2 infects microglia *in vitro* and in a murine model, both of which produce pro-inflammatory cytokines (32). Multiple strains of IAV can infect murine and human microglia and produce cytokines similarly to other RNA viruses, including IL-6 and MCP-1 (98, 102). HIV-1 infects iPSC-derived microglia combined with brain organoids that cause persistent type 1 interferon signaling (116). Some RNA viruses, such as JEV, SARS-CoV-2, IAV, and HIV-1, lead to apoptosis of these cells or surrounding neurons (32, 38, 39, 98, 117). Microglia have been identified as another potential reservoir for infection in ZIKV and JEV due to *in vitro* data indicating long-term microglial infection and the capacity for microglia to spread infection to other CNS cell types (23, 40, 41). Microglia are a major source of replicating HIV-1 in the CNS (118). HIV-1 has been found to infect microglia in cell culture and in individuals who were infected with HIV-1 (110, 112, 118). Microglia can also be persistently infected with HIV-1 *in vitro*, suggesting that microglia may be a reservoir for infection (119, 120). There is evidence that the rubella virus preferentially infects microglia in CNS organoid models (82).

### DNA viruses

There is relatively limited information on DNA viral infection of microglia. Both HCMV and MPXV infect microglia in cell culture (138, 170). HCMV has also been identified in morphologically identified monocyte-derived macrophages/microglial cells within human brain cultures (141). HCMV-infected microglia produced chemokines and pro-inflammatory cytokines, such as tumor necrosis factor-alpha (TNF-α) (140). MPXV infection of primary human microglia was less productive than infection of astrocytes. MPXV-infected microglia had higher gene expression for genes that restrict MPXV replication, which could explain less virus production (170). Parvovirus B19 has been identified within activated microglia in post-mortem fetal brain tissue, but the role in pathogenesis is unclear (154, 155).

While the primary function of microglia is to detect and respond to infection, viral replication within the microglia itself may lead to pathology. This may be through loss of function or through persistence of unintended neuroinflammation that causes damage to neighboring cells. Additional studies examining how microglia act as a viral reservoir or how infected microglia return to their resting homeostatic state after an infectious insult could further our understanding of their role in neuropathogenesis.

## OLIGODENDROCYTES

Oligodendrocytes insulate the axons of neurons through the formation of a myelin sheath. This sheath protects neurons and increases the speed of action potential transit (203). Damage to oligodendrocytes and/or the myelin sheath can disrupt neuron signaling and harm the neurons, causing severe disease. Similarly, some viruses induce demyelination by damaging oligodendrocytes that produce the myelin sheath. Much of our understanding of oligodendrocyte infection in viral neuropathogenesis is from non-human pathogens, such as Theiler’s murine encephalomyelitis virus (TMEV) and murine hepatitis virus (MHV); however, these are outside of the scope of this review (204, 205).

The lack of studies investigating human viruses and oligodendrocytes may be, in part, due to challenges with modeling oligodendrocytes *in vitro* and the lack of reliable oligodendrocyte cell lines (206). Primary oligodendrocytes need neurons to exist and function properly, indicating that a co-culture system is required for *in vitro* study. As such, human multicellular organoid models may be particularly useful for studying the role of oligodendrocytes in viral pathogenesis.

### RNA viruses

Most work to date investigating viral infection of oligodendrocytes has evaluated the impact of infection on cell death. TBEV readily infects oligodendrocytes in mixed neuronal/glial cell cultures, although oligodendrocytes did not die during infection (45). HIV-1 can productively infect primary isolated oligodendrocytes *in vitro*. In the same study, infected microglia were able to transfer viral infection to oligodendrocytes (121). HIV-1 DNA has also been discovered in brain oligodendrocytes upon autopsy of patients with AIDS (122). EV-D68 may also infect oligodendrocytes or oligodendrocyte precursor cells in human spinal cord organoids based on single-cell sequencing data (94). MeV readily infects human oligodendroglioma cells and rat oligodendrocytes. This aligns with MeV-infected oligodendrocytes seen in SSPE caused by MeV infection (107). Multiple RNA viruses infect oligodendrocytes and then cause apoptotic cell death, including rubella virus in rat oligodendrocytes, ZIKV in a perinatal mouse model, and poliovirus in a mixed mouse primary cell culture model (69, 83, 95). There is strong evidence that RNA viruses have the capacity to infect oligodendrocytes, but significant mechanistic work remains to expand conclusions beyond the potential for oligodendrocyte cell death.

### DNA viruses

Multiple DNA viruses have been shown to have oligodendrocyte tropism: HSV-1, VZV, parvovirus B19, and JCPyV. HSV-1 infects primary human oligodendrocyte cultures and an oligodendrocyte cell line (131, 132). Oligodendrocyte infection may contribute to HSV-1 replication by trafficking microvesicles that contain HSV-1 virions and express myelin and lymphocyte protein (MAL) (133, 134). VZV was identified in oligodendrocytes of patients with herpes zoster myelitis (152). Parvovirus B19 was primarily found in oligodendrocytes of post-mortem brain tissue (154).

Perhaps the best evidence for oligodendrocyte infection leading to disease is for JCPyV. JCPyV infects oligodendrocytes *in vitro* and has been found in patient oligodendrocytes and oligodendrogliomas (158, 162). The protein Akt, involved in cell growth and proliferation, is needed for oligodendrocyte infection, and active glial cell proliferation benefits JCPyV replication and spread (162). Apoptotic oligodendrocytes have been found in patients with PML, possibly induced by the viral protein agnoprotein (163, 164). Another viral protein, T-antigen, antagonizes apoptosis through upregulation of survivin, which was originally found to be upregulated in infected clinical samples (165, 166). It is thought that survivin is needed to complete the JCPyV replication cycle and that the lack of survivin increases cell death.

Multiple viruses can infect oligodendrocytes, most likely resulting in oligodendrocyte death. This leads to devastating effects on neuron protection and signal transmission, resulting in neuron damage and loss of function. As *in vitro* cellular models for studying human oligodendrocytes improve, we expect that their role in neuropathogenesis after viral infection will become more recognized.

## NEURAL PROGENITOR CELLS

Neural progenitor cells (NPCs) are pluripotent CNS cells that differentiate into neuronal or glial cells (207). They play a critical role in neurogenesis and human brain development, with NPC dysfunction linked to various developmental disorders (208). Viral infection can lead to changes in NPC proliferation, altering their capacity to differentiate properly, and cause NPC death, which limits developmental potential (9). NPCs are critical for early neurodevelopment, but they are present throughout life to help maintain the neuronal and glial populations as cell turnover occurs (209).

NPCs can be studied in human and animal tissues, primary cells, cell lines, and organoids. Primary NPCs and iPSC-derived NPCs are often used for monoculture studies, but some human cell lines do exist. Human cell lines have some physiologic variability, such as NPC, and after differentiation into other cell types (210). Due to the similarity to stem cells, NPCs are present in CNS organoids early during the differentiation protocols. These models may be particularly beneficial for studying viral infection of NPCs and its impacts on cell survival and development in the context of other cell types.

### RNA viruses

While multiple flaviviruses can infect NPCs, ZIKV has been the most studied, perhaps due to its role in congenital infection leading to microcephaly. ZIKV infects human and murine NPCs and inhibits NPC proliferation and differentiation (70–73). *In vivo* work has shown that ZIKV-infected NPCs have disrupted differentiation, leading to microcephaly in mice (74). This is in part due to ZIKV activation of the NOTCH pathway, which is involved in cellular proliferation and differentiation (75). ZIKV infection of NPCs allows high-titer viral replication with little innate immune response in fetal brain autopsy samples (76). Infected NPCs trigger programmed cellular death through apoptosis or pyroptosis (76–78). JEV infects NPCs, decreasing proliferation and differentiation *in vitro*, where it can also cause NPC cell death through increasing cellular stress (42–44). WNV infects NPCs, causing significant cytopathic effects *in vitro* (56). Flavivirus infection of NPCs can result in cell death, which halts differentiation required for continued brain development. This contributes to microcephaly and developmental disorders common with multiple flavivirus infections (211).

Other RNA viruses with NPC tropism include rubella virus, SARS-CoV-2, IAV, HIV-1, and EV-A71. Rubella virus antigen has been found in NPCs of fetal and neonatal clinical samples (84). SARS-CoV-2 can infect NPCs *in vitro* and within brain organoids, and infected NPCs have been found to succumb to cell death (33, 34). IAV infects NPCs *in vitro,* which induces production of pro-inflammatory cytokines (20). HIV-1 has been found in NPCs of pediatric brain tissue (123). HIV-1 infects NPCs *in vitro,* and is considered a potential reservoir of HIV-1 in the brain (124, 125). EV-A71 infects NPCs *in vitro* although not as productively as other CNS cell types (88). NPC metabolic testing revealed that significant upregulation of glucose metabolic pathways occurred during EV-A71 infection (89). It appears the RNA viruses can infect NPCs with varying consequences that could contribute to neuropathogenesis.

### DNA viruses

MPXV and JCPyV have NPC tropism as well as HCMV and HSV. MPXV productively infects NPCs in monocultures and neural organoids (21, 169). JCPyV can infect glial progenitor cells, a type of neural progenitor cell, and contribute to loss of differentiation both *in vivo* and *in vitro* (157, 167, 168). HSV-1 infects NPCs *in vitro* and causes both acute lytic and latent infection (135, 136). HSV-1 infection also alters NPC gene expression changes, causing dysregulation of proliferation and differentiation (137). HCMV readily infects NPCs and dramatically alters gene expression to lose pluripotency *in vitro* (22, 142–144). Viral protein immediate early 1 (IE1) mediates part of this loss through interactions with STAT3 (145). Viral microRNAs may also be involved with NPC regulation during HCMV infection (146). NPCs undergo apoptosis upon HCMV infection (147). DNA viruses infect and cause the death of NPCs, and they cause NPC dysfunction through gene expression changes, halting NPC differentiation.

Multiple viruses that infect NPCs cause neuropathogenesis through cell death or disruption of differentiation into other neural cells, initiating enduring consequences on the function of the developing brain and spinal cord.

## CONCLUDING REMARKS

Neurotropic viral infections cause significant morbidity and mortality. Currently, most neurotropic viral illnesses lack specific therapeutics or preventative vaccines. This review examines the current literature regarding the role of non-neuronal cells in viral neuropathogenesis with a focus on astrocytes, microglia, oligodendrocytes, and NPCs. While our scope was limited to human viruses, it is evident that many neurotropic viruses target glial cells (Fig. 1, Table 1). However, this nascent field requires additional inquiry to assess the distinct role of glial cell infection in neuroprotection and neuropathogenesis. This field is emerging due to the recent increase in complexity of human model systems of the human brain and spinal cord. Current studies have provided foundational knowledge about basic cellular dysfunction, cellular death, and innate immune response triggered by viral pathogens. Further investigations exploring the mechanisms of how infection of these critical cells contributes to neuropathogenesis are greatly needed to advance this field. As immunopathogenesis and viral dissemination are critical components of understanding CNS viral infections, there is a continued need for complementary animal models for the study of CNS pathogens. Future studies require increased complexity of multicellular human models that incorporate both innate and adaptive immune components to more accurately recapitulate viral neuropathogenesis. Ongoing innovation provides an opportunity to revolutionize the mechanistic understanding of neurovirulence caused by glial cell infection. Ultimately, uncovering convergent viral strategies of neuropathogenesis will lead to improved development of targeted therapeutics.

## References

[1] Ludlow M, Kortekaas J, Herden C, Hoffmann B, Tappe D, Trebst C, Griffin DE, Brindle HE, Solomon T, Brown AS, van Riel D, Wolthers KC, Pajkrt D, Wohlsein P, Martina BEE, Baumgärtner W, Verjans GM, Osterhaus ADME. 2016. Neurotropic virus infections as the cause of immediate and delayed neuropathology. Acta Neuropathol 131:159–184. doi:10.1007/s00401-015-1511-3 26659576 PMC4713712

[2] Abdullahi AM, Sarmast ST, Jahan N. 2020. Viral infections of the central nervous system in children: a systematic review. Cureus 12:e11174. doi:10.7759/cureus.11174 33262911 PMC7689876

[3] Kennedy PGE. 2021. An overview of viral infections of the nervous system in the immunosuppressed. J Neurol 268:3026–3030. doi:10.1007/s00415-020-10265-z 33048220 PMC7552955

[4] Shereen MA, Bashir N, Su R, Liu F, Wu K, Luo Z, Wu J. 2021. Zika virus dysregulates the expression of astrocytic genes involved in neurodevelopment. PLoS Negl Trop Dis 15:e0009362. doi:10.1371/journal.pntd.0009362 33891593 PMC8099136

[5] Orr N, Steinman L. 2025. Epstein–Barr virus and the immune microenvironment in multiple sclerosis: Insights from high-dimensional brain tissue imaging. Proc Natl Acad Sci USA 122. doi:10.1073/pnas.2425670122 PMC11929469 40063794

[6] Blázquez AB, Saiz JC. 2016. Neurological manifestations of Zika virus infection. World J Virol 5:135–143. doi:10.5501/wjv.v5.i4.135 27878100 PMC5105046

[7] Mohabbat A, Bannazadeh Baghi H. 2025. Chronic neuroplasticity changes following neurotropic viral infection: mechanisms and implications. Cell Mol Neurobiol 45:94. doi:10.1007/s10571-025-01622-5 41186612 PMC12586820

[8] Allen NJ, Lyons DA. 2018. Glia as architects of central nervous system formation and function. Science 362:181–185. doi:10.1126/science.aat0473 30309945 PMC6292669

[9] Kamte YS, Chandwani MN, Michaels AC, O’Donnell LA. 2021. Neural stem cells: what happens when they go viral? Viruses 13:1468. doi:10.3390/v13081468 34452333 PMC8402908

[10] Blackhurst BM, Funk KE. 2023. Molecular and cellular mechanisms underlying neurologic manifestations of mosquito-borne flavivirus infections. Viruses 15:2200. doi:10.3390/v15112200 38005878 PMC10674799

[11] Jha HC, Mehta D, Lu J, El-Naccache D, Shukla SK, Kovacsics C, Kolson D, Robertson ES. 2015. Gammaherpesvirus infection of human neuronal cells. mBio 6:e01844-15. doi:10.1128/mBio.01844-15 26628726 PMC4669387

[12] Huang HI, Shih SR. 2015. Neurotropic enterovirus infections in the central nervous system. Viruses 7:6051–6066. doi:10.3390/v7112920 26610549 PMC4664993

[13] von Bartheld CS, Bahney J, Herculano‐Houzel S. 2016. The search for true numbers of neurons and glial cells in the human brain: a review of 150 years of cell counting. J of Comparative Neurology 524:3865–3895. doi:10.1002/cne.24040 PMC5063692 27187682

[14] Koyuncu OO, Hogue IB, Enquist LW. 2013. Virus infections in the nervous system. Cell Host Microbe 13:379–393. doi:10.1016/j.chom.2013.03.010 23601101 PMC3647473

[15] Zeng Z, Miao N, Sun T. 2018. Revealing cellular and molecular complexity of the central nervous system using single cell sequencing. Stem Cell Res Ther 9:234. doi:10.1186/s13287-018-0985-z 30213269 PMC6137869

[16] Soorajkumar A, Balan B, Nassir N, Akter H, Shahin Z, Berdiev BK, Woodbury-Smith M, Khalil R, Cisse B, Benamer HTS, Uddin M. 2025. Mapping human brain cell type origin and diseases through single-cell transcriptomics. Transl Psychiatry 15:349. doi:10.1038/s41398-025-03562-6 41053013 PMC12501371

[17] Wang Y, Peng D, Li M, Yao M, Li T, Li S, Qiu HJ, Li LF. 2025. Organoids: physiologically relevant ex vivo models for viral disease research. J Virol 99:e0113225. doi:10.1128/jvi.01132-25 40879383 PMC12456137

[18] D’Aiuto L, Caldwell JK, Wallace CT, Grams TR, Wesesky MA, Wood JA, Watkins SC, Kinchington PR, Bloom DC, Nimgaonkar VL. 2022. The impaired neurodevelopment of human neural rosettes in HSV-1-infected early brain organoids. Cells 11:3539. doi:10.3390/cells11223539 36428968 PMC9688774

[19] Garcez PP, Loiola EC, Madeiro da Costa R, Higa LM, Trindade P, Delvecchio R, Nascimento JM, Brindeiro R, Tanuri A, Rehen SK. 2016. Zika virus impairs growth in human neurospheres and brain organoids. Science 352:816–818. doi:10.1126/science.aaf6116 27064148

[20] Pringproa K, Rungsiwiwut R, Tantilertcharoen R, Praphet R, Pruksananonda K, Baumgärtner W, Thanawongnuwech R. 2015. Tropism and induction of cytokines in human embryonic-stem cells-derived neural progenitors upon inoculation with highly- pathogenic avian H5N1 influenza virus. PLoS One 10:e0135850. doi:10.1371/journal.pone.0135850 26274828 PMC4537284

[21] Chailangkarn T, Teeravechyan S, Attasombat K, Thaweerattanasinp T, Sunchatawirul K, Suwanwattana P, Pongpirul K, Jongkaewwattana A. 2022. Monkeypox virus productively infects human induced pluripotent stem cell-derived astrocytes and neural progenitor cells. J Infect 85:702–769. doi:10.1016/j.jinf.2022.10.016 PMC9580251 36272454

[22] D’Aiuto L, Di Maio R, Heath B, Raimondi G, Milosevic J, Watson AM, Bamne M, Parks WT, Yang L, Lin B, Miki T, Mich-Basso JD, Arav-Boger R, Sibille E, Sabunciyan S, Yolken R, Nimgaonkar V. 2012. Human induced pluripotent stem cell-derived models to investigate human cytomegalovirus infection in neural cells. PLoS One 7:e49700. doi:10.1371/journal.pone.0049700 23209593 PMC3507916

[23] Muffat J, Li Y, Omer A, Durbin A, Bosch I, Bakiasi G, Richards E, Meyer A, Gehrke L, Jaenisch R. 2018. Human induced pluripotent stem cell-derived glial cells and neural progenitors display divergent responses to Zika and dengue infections. Proc Natl Acad Sci USA 115:7117–7122. doi:10.1073/pnas.1719266115 29915057 PMC6142255

[24] Haverty R, McCormack J, Evans C, Purves K, O’Reilly S, Gautier V, Rochfort K, Fabre A, Fletcher NF. 2024. SARS-CoV-2 infects neurons, astrocytes, choroid plexus epithelial cells and pericytes of the human central nervous system in vitro. J Gen Virol 105. doi:10.1099/jgv.0.002009 PMC11317966 38995681

[25] Andrews MG, Mukhtar T, Eze UC, Simoneau CR, Ross J, Parikshak N, Wang S, Zhou L, Koontz M, Velmeshev D, et al.. 2022. Tropism of SARS-CoV-2 for human cortical astrocytes. Proc Natl Acad Sci USA 119. doi:10.1073/pnas.2122236119 PMC9335272 35858406

[26] Robinson KF, Ahiya AI, Richner JM, Lutz SE. 2025. SARS-CoV-2 infection influences Wnt/β-catenin pathway components in astrocytes. Pathogens 14:994. doi:10.3390/pathogens14100994 41156605 PMC12567431

[27] Bhide K, Slavikova M, Talpasova L, Kuckova K, Klempa B, Tyagi P, Bhide M. 2025. Comprehensive mapping of the signaling events evoked by SARS-CoV-2 variants delta and omicron in human astrocytes. Sci Rep 15:36897. doi:10.1038/s41598-025-20851-8 41125670 PMC12546740

[28] Colinet M, Chiver I, Bonafina A, Masset G, Almansa D, Di Valentin E, Twizere J-C, Nguyen L, Espuny-Camacho I. 2025. SARS-CoV2 infection triggers inflammatory conditions and astrogliosis-related gene expression in long-term human cortical organoids. Stem Cells 43:sxaf010. doi:10.1093/stmcls/sxaf010 40103011 PMC12121356

[29] Gerzanich V, Zhang C, Zhang J, Sallapalli BT, Pei S, Nasr M, Tosun C, Zhang Y, Tang Q, Simard JM, Zhao RY. 2025. COVID-19-associated neuroinflammation and astrocyte death in the brain linked to ORF3a-induced activation of Sur1-mediated ion channels. mBio 16:e0201225. doi:10.1128/mbio.02012-25 40802282 PMC12421821

[30] Kong W, Montano M, Corley MJ, Helmy E, Kobayashi H, Kinisu M, Suryawanshi R, Luo X, Royer LA, Roan NR, Ott M, Ndhlovu LC, Greene WC. 2022. Neuropilin-1 mediates SARS-CoV-2 infection of astrocytes in brain organoids, inducing inflammation leading to dysfunction and death of neurons. mBio 13:e0230822. doi:10.1128/mbio.02308-22 36314791 PMC9765283

[31] Segabinazi E, Tocantins FR, Glaser T, Maglio T, Oliveira NC, Castanha AG, Baldino Russo F, Corrêa Leite PE, Brito A, Molina CV, Prado Paludo G, Souza R de O, Alves SRM, Cunha MDP, Ulrich H, Durigon EL, Minoprio P, Beltrão-Braga PCB. 2025. Astroglia-mediated neuroinflammation as a putative mechanism of neurological outcomes in COVID-19? Insights from a Brazilian cohort. Brain Behav Immun Health 49:101115. doi:10.1016/j.bbih.2025.101115 41141865 PMC12547199

[32] Jeong GU, Lyu J, Kim KD, Chung YC, Yoon GY, Lee S, Hwang I, Shin WH, Ko J, Lee JY, Kwon YC. 2022. SARS-CoV-2 infection of microglia elicits proinflammatory activation and apoptotic cell death. Microbiol Spectr 10:e0109122. doi:10.1128/spectrum.01091-22 35510852 PMC9241873

[33] Zhang B-Z, Chu H, Han S, Shuai H, Deng J, Hu Y-F, Gong H-R, Lee AC-Y, Zou Z, Yau T, Wu W, Hung IF-N, Chan JF-W, Yuen K-Y, Huang J-D. 2020. SARS-CoV-2 infects human neural progenitor cells and brain organoids. Cell Res 30:928–931. doi:10.1038/s41422-020-0390-x 32753756 PMC7399356

[34] Mesci P, de Souza JS, Martin-Sancho L, Macia A, Saleh A, Yin X, Snethlage C, Adams JW, Avansini SH, Herai RH, Almenar-Queralt A, Pu Y, Szeto RA, Goldberg G, Bruck PT, Papes F, Chanda SK, Muotri AR. 2022. SARS-CoV-2 infects human brain organoids causing cell death and loss of synapses that can be rescued by treatment with Sofosbuvir. PLoS Biol 20:e3001845. doi:10.1371/journal.pbio.3001845 36327326 PMC9632769

[35] Siva Venkatesh IP, Bhaskar M, Basu A. 2022. Japanese encephalitis viral infection modulates proinflammatory cyto/chemokine profile in primary astrocyte and cell line of astrocytic origin. Metab Brain Dis 37:1487–1502. doi:10.1007/s11011-022-00991-w 35486209

[36] Mishra MK, Kumawat KL, Basu A. 2008. Japanese encephalitis virus differentially modulates the induction of multiple pro-inflammatory mediators in human astrocytoma and astroglioma cell-lines. Cell Biol Int 32:1506–1513. doi:10.1016/j.cellbi.2008.08.020 18801452

[37] Chen C-J, Ou Y-C, Chang C-Y, Pan H-C, Liao S-L, Raung S-L, Chen S-Y. 2011. TNF-α and IL-1β mediate Japanese encephalitis virus-induced RANTES gene expression in astrocytes. Neurochem Int 58:234–242. doi:10.1016/j.neuint.2010.12.009 21167894

[38] Chen C-J, Ou Y-C, Lin S-Y, Raung S-L, Liao S-L, Lai C-Y, Chen S-Y, Chen J-H. 2010. Glial activation involvement in neuronal death by Japanese encephalitis virus infection. Journal of General Virology 91:1028–1037. doi:10.1099/vir.0.013565-0 20007359

[39] Myint KSA, Kipar A, Jarman RG, Gibbons RV, Perng GC, Flanagan B, Mongkolsirichaikul D, Van Gessel Y, Solomon T. 2014. Neuropathogenesis of Japanese encephalitis in a primate model. PLoS Negl Trop Dis 8:e2980. doi:10.1371/journal.pntd.0002980 25102067 PMC4125110

[40] Thongtan T, Cheepsunthorn P, Chaiworakul V, Rattanarungsan C, Wikan N, Smith DR. 2010. Highly permissive infection of microglial cells by Japanese encephalitis virus: a possible role as a viral reservoir. Microbes Infect 12:37–45. doi:10.1016/j.micinf.2009.09.013 19786116

[41] Lannes N, Garcia-Nicolàs O, Démoulins T, Summerfield A, Filgueira L. 2019. CX3CR1-CX3CL1-dependent cell-to-cell Japanese encephalitis virus transmission by human microglial cells. Sci Rep 9:4833. doi:10.1038/s41598-019-41302-1 30886214 PMC6423114

[42] Das S, Basu A. 2008. Japanese encephalitis virus infects neural progenitor cells and decreases their proliferation. J Neurochem 106:1624–1636. doi:10.1111/j.1471-4159.2008.05511.x 18540995

[43] Ariff IM, Thounaojam MC, Das S, Basu A. 2013. Japanese encephalitis virus infection alters both neuronal and astrocytic differentiation of neural stem/progenitor cells. J Neuroimmune Pharmacol 8:664–676. doi:10.1007/s11481-013-9455-7 23546886

[44] Mukherjee S, Singh N, Sengupta N, Fatima M, Seth P, Mahadevan A, Shankar SK, Bhattacharyya A, Basu A. 2018. Japanese encephalitis virus induces human neural stem/progenitor cell death by elevating GRP78, PHB and hnRNPC through ER stress. Cell Death Dis 8:e2556–e2556. doi:10.1038/cddis.2016.394 PMC5386351 28102850

[45] Fares M, Cochet-Bernoin M, Gonzalez G, Montero-Menei CN, Blanchet O, Benchoua A, Boissart C, Lecollinet S, Richardson J, Haddad N, Coulpier M. 2020. Pathological modeling of TBEV infection reveals differential innate immune responses in human neurons and astrocytes that correlate with their susceptibility to infection. J Neuroinflammation 17:76. doi:10.1186/s12974-020-01756-x 32127025 PMC7053149

[46] Potokar M, Korva M, Jorgačevski J, Avšič-Županc T, Zorec R. 2014. Tick-borne encephalitis virus infects rat astrocytes but does not affect their viability. PLoS One 9:e86219. doi:10.1371/journal.pone.0086219 24465969 PMC3896472

[47] Lindqvist R, Mundt F, Gilthorpe JD, Wölfel S, Gekara NO, Kröger A, Överby AK. 2016. Fast type I interferon response protects astrocytes from flavivirus infection and virus-induced cytopathic effects. J Neuroinflammation 13:277. doi:10.1186/s12974-016-0748-7 27776548 PMC5078952

[48] Pranclova V, Nedvedova L, Kotounova E, Hönig V, Dvorakova M, Davidkova M, Bily T, Vancova M, Ruzek D, Palus M. 2024. Unraveling the role of human microglia in tick-borne encephalitis virus infection: insights into neuroinflammation and viral pathogenesis. Microbes Infect 26:105383. doi:10.1016/j.micinf.2024.105383 38942136

[49] Potokar M, Jorgačevski J, Zorec R. 2019. Astrocytes in flavivirus infections. Int J Mol Sci 20:691. doi:10.3390/ijms20030691 30736273 PMC6386967

[50] van Marle G, Antony J, Ostermann H, Dunham C, Hunt T, Halliday W, Maingat F, Urbanowski MD, Hobman T, Peeling J, Power C. 2007. West nile virus-induced neuroinflammation: glial infection and capsid protein-mediated neurovirulence. J Virol 81:10933–10949. doi:10.1128/JVI.02422-06 17670819 PMC2045515

[51] Blakely PK, Kleinschmidt-DeMasters BK, Tyler KL, Irani DN. 2009. Disrupted glutamate transporter expression in the spinal cord with acute flaccid paralysis caused by west nile virus infection. J Neuropathol Exp Neurol 68:1061–1072. doi:10.1097/NEN.0b013e3181b8ba14 19918118 PMC2854566

[52] Quick ED, Leser JS, Clarke P, Tyler KL. 2014. Activation of intrinsic immune responses and microglial phagocytosis in an ex vivo spinal cord slice culture model of West Nile virus infection. J Virol 88:13005–13014. doi:10.1128/JVI.01994-14 25165111 PMC4249089

[53] Cheeran MC-J, Hu S, Sheng WS, Rashid A, Peterson PK, Lokensgard JR. 2005. Differential responses of human brain cells to West Nile virus infection. J Neurovirol 11:512–524. doi:10.1080/13550280500384982 16338745

[54] Hussmann KL, Fredericksen BL. 2014. Differential induction of CCL5 by pathogenic and non-pathogenic strains of West Nile virus in brain endothelial cells and astrocytes. J Gen Virol 95:862–867. doi:10.1099/vir.0.060558-0 24413421 PMC3973477

[55] Hussmann KL, Vandergaast R, Zheng K, Hoover LI, Fredericksen BL. 2014. Structural proteins of West Nile virus are a major determinant of infectious particle production and fitness in astrocytes. J Gen Virol 95:1991–2003. doi:10.1099/vir.0.065474-0 24920724 PMC4135089

[56] Riccetti S, Sinigaglia A, Desole G, Nowotny N, Trevisan M, Barzon L. 2020. Modelling west nile virus and usutu virus pathogenicity in human neural stem cells. Viruses 12:882. doi:10.3390/v12080882 32806715 PMC7471976

[57] Stefanik M, Formanova P, Bily T, Vancova M, Eyer L, Palus M, Salat J, Braconi CT, Zanotto PM de A, Gould EA, Ruzek D. 2018. Characterisation of Zika virus infection in primary human astrocytes. BMC Neurosci 19:5. doi:10.1186/s12868-018-0407-2 29463209 PMC5820785

[58] Rubio-Hernández EI, Comas-García M, Coronado-Ipiña MA, Colunga-Saucedo M, González Sánchez HM, Castillo CG. 2023. Astrocytes derived from neural progenitor cells are susceptible to Zika virus infection. PLoS One 18:e0283429. doi:10.1371/journal.pone.0283429 36989308 PMC10057746

[59] Ledur PF, Karmirian K, Pedrosa C da SG, Souza LRQ, Assis-de-Lemos G, Martins TM, Ferreira J de CCG, de Azevedo Reis GF, Silva ES, Silva D, Salerno JA, Ornelas IM, Devalle S, Madeiro da Costa RF, Goto-Silva L, Higa LM, Melo A, Tanuri A, Chimelli L, Murata MM, Garcez PP, Filippi-Chiela EC, Galina A, Borges HL, Rehen SK. 2020. Zika virus infection leads to mitochondrial failure, oxidative stress and DNA damage in human iPSC-derived astrocytes. Sci Rep 10:1218. doi:10.1038/s41598-020-57914-x 31988337 PMC6985105

[60] Veilleux C, Eugenin EA. 2023. Mechanisms of Zika astrocyte infection and neuronal toxicity. NeuroImmune Pharm Ther 2:5–18. doi:10.1515/nipt-2022-0014 37027343 PMC10070016

[61] Monel B, Compton AA, Bruel T, Amraoui S, Burlaud-Gaillard J, Roy N, Guivel-Benhassine F, Porrot F, Génin P, Meertens L, Sinigaglia L, Jouvenet N, Weil R, Casartelli N, Demangel C, Simon-Lorière E, Moris A, Roingeard P, Amara A, Schwartz O. 2017. Zika virus induces massive cytoplasmic vacuolization and paraptosis-like death in infected cells. EMBO J 36:1653–1668. doi:10.15252/embj.201695597 28473450 PMC5470047

[62] Huang Y, Li Y, Zhang H, Zhao R, Jing R, Xu Y, He M, Peer J, Kim YC, Luo J, Tong Z, Zheng J. 2018. Zika virus propagation and release in human fetal astrocytes can be suppressed by neutral sphingomyelinase-2 inhibitor GW4869. Cell Discov 4:19. doi:10.1038/s41421-018-0017-2 29707233 PMC5913238

[63] Simonin Y, Loustalot F, Desmetz C, Foulongne V, Constant O, Fournier-Wirth C, Leon F, Molès J-P, Goubaud A, Lemaitre J-M, Maquart M, Leparc-Goffart I, Briant L, Nagot N, Van de Perre P, Salinas S. 2016. Zika virus strains potentially display different infectious profiles in human neural cells. EBioMedicine 12:161–169. doi:10.1016/j.ebiom.2016.09.020 27688094 PMC5078617

[64] Hamel R, Ferraris P, Wichit S, Diop F, Talignani L, Pompon J, Garcia D, Liégeois F, Sall AA, Yssel H, Missé D. 2017. African and Asian Zika virus strains differentially induce early antiviral responses in primary human astrocytes. Infection, Genetics and Evolution 49:134–137. doi:10.1016/j.meegid.2017.01.015 28095299

[65] Meertens L, Labeau A, Dejarnac O, Cipriani S, Sinigaglia L, Bonnet-Madin L, Le Charpentier T, Hafirassou ML, Zamborlini A, Cao-Lormeau VM, Coulpier M, Missé D, Jouvenet N, Tabibiazar R, Gressens P, Schwartz O, Amara A. 2017. Axl mediates zika virus entry in human glial cells and modulates innate immune responses. Cell Rep 18:324–333. doi:10.1016/j.celrep.2016.12.045 28076778

[66] Ojha CR, Rodriguez M, Karuppan MKM, Lapierre J, Kashanchi F, El-Hage N. 2019. Toll-like receptor 3 regulates Zika virus infection and associated host inflammatory response in primary human astrocytes. PLoS One 14:e0208543. doi:10.1371/journal.pone.0208543 30735502 PMC6368285

[67] Diop F, Vial T, Ferraris P, Wichit S, Bengue M, Hamel R, Talignani L, Liegeois F, Pompon J, Yssel H, Marti G, Missé D. 2018. Zika virus infection modulates the metabolomic profile of microglial cells. PLoS One 13:e0206093. doi:10.1371/journal.pone.0206093 30359409 PMC6201926

[68] Lum F-M, Low DKS, Fan Y, Tan JJL, Lee B, Chan JKY, Rénia L, Ginhoux F, Ng LFP. 2017. Zika virus infects human fetal brain microglia and induces inflammation. Clin Infect Dis 64:914–920. doi:10.1093/cid/ciw878 28362944

[69] Schultz V, Barrie JA, Donald CL, Crawford CL, Mullin M, Anderson TJ, Solomon T, Barnett SC, Linington C, Kohl A, Willison HJ, Edgar JM. 2021. Oligodendrocytes are susceptible to Zika virus infection in a mouse model of perinatal exposure: implications for CNS complications. Glia 69:2023–2036. doi:10.1002/glia.24010 33942402 PMC9216243

[70] Tang H, Hammack C, Ogden SC, Wen Z, Qian X, Li Y, Yao B, Shin J, Zhang F, Lee EM, Christian KM, Didier RA, Jin P, Song H, Ming G. 2016. Zika virus infects human cortical neural progenitors and attenuates their growth. Cell Stem Cell 18:587–590. doi:10.1016/j.stem.2016.02.016 26952870 PMC5299540

[71] Brault J-B, Khou C, Basset J, Coquand L, Fraisier V, Frenkiel M-P, Goud B, Manuguerra J-C, Pardigon N, Baffet AD. 2016. Comparative analysis between flaviviruses reveals specific neural stem cell tropism for zika virus in the mouse developing neocortex. EBioMedicine 10:71–76. doi:10.1016/j.ebiom.2016.07.018 27453325 PMC5006693

[72] Devhare P, Meyer K, Steele R, Ray RB, Ray R. 2017. Zika virus infection dysregulates human neural stem cell growth and inhibits differentiation into neuroprogenitor cells. Cell Death Dis 8:e3106–e3106. doi:10.1038/cddis.2017.517 29022904 PMC5682681

[73] Li H, Saucedo-Cuevas L, Regla-Nava JA, Chai G, Sheets N, Tang W, Terskikh AV, Shresta S, Gleeson JG. 2016. Zika virus infects neural progenitors in the adult mouse brain and alters proliferation. Cell Stem Cell 19:593–598. doi:10.1016/j.stem.2016.08.005 27545505 PMC5097023

[74] Li C, Xu D, Ye Q, Hong S, Jiang Y, Liu X, Zhang N, Shi L, Qin C-F, Xu Z. 2016. Zika virus disrupts neural progenitor development and leads to microcephaly in mice. Cell Stem Cell 19:120–126. doi:10.1016/j.stem.2016.04.017 27179424

[75] Ferraris P, Cochet M, Hamel R, Gladwyn-Ng I, Alfano C, Diop F, Garcia D, Talignani L, Montero-Menei CN, Nougairède A, Yssel H, Nguyen L, Coulpier M, Missé D. 2019. Zika virus differentially infects human neural progenitor cells according to their state of differentiation and dysregulates neurogenesis through the Notch pathway. Emerging Microbes & Infections 8:1003–1016. doi:10.1080/22221751.2019.1637283 31282298 PMC6691766

[76] Stokes C, Whitmore LS, Moreno D, Malhotra K, Tisoncik-Go J, Tran E, Wren N, Glass IA, Young JE, Gale M Jr. 2025. The human neural cell atlas of Zika virus infection in developing brain tissue. Cell Reports Medicine 6:102189. doi:10.1016/j.xcrm.2025.102189 40532660 PMC12208335

[77] Souza BSF, Sampaio GLA, Pereira CS, Campos GS, Sardi SI, Freitas LAR, Figueira CP, Paredes BD, Nonaka CKV, Azevedo CM, Rocha VPC, Bandeira AC, Mendez-Otero R, Dos Santos RR, Soares MBP. 2016. Zika virus infection induces mitosis abnormalities and apoptotic cell death of human neural progenitor cells. Sci Rep 6:39775. doi:10.1038/srep39775 28008958 PMC5180086

[78] He Z, An S, Chen J, Zhang S, Tan C, Yu J, Ye H, Wu Y, Yuan J, Wu J, Zhu X, Li M. 2020. Neural progenitor cell pyroptosis contributes to Zika virus-induced brain atrophy and represents a therapeutic target. Proc Natl Acad Sci USA 117:23869–23878. doi:10.1073/pnas.2007773117 32907937 PMC7519283

[79] Desmond MM, Wilson GS, Melnick JL, Singer DB, Zion TE, Rudolph AJ, Pineda RG, Ziai M-H, Blattner RJ. 1967. Congenital rubella encephalitis. course and early sequelae. J Pediatr 71:311–331. doi:10.1016/s0022-3476(67)80291-9 6034785

[80] Chantler JK, Smyrnis L, Tai G. 1995. Selective infection of astrocytes in human glial cell cultures by rubella virus. Lab Invest 72:334–340. 7898052

[81] Atkins GJ, Mooney DA, Fahy DA, Ng SH, Sheahan BJ. 1991. Multiplication of rubella and measles viruses in primary rat neural cell cultures: relevance to a postulated triggering mechanism for multiple sclerosis. Neuropathol Appl Neurobiol 17:299–308. doi:10.1111/j.1365-2990.1991.tb00727.x 1719441 PMC7194296

[82] Popova G, Retallack H, Kim CN, Wang A, Shin D, DeRisi JL, Nowakowski T. 2023. Rubella virus tropism and single-cell responses in human primary tissue and microglia-containing organoids. eLife 12:RP87696. doi:10.7554/eLife.87696 37470786 PMC10370260

[83] Domegan LM, Atkins GJ. 2002. Apoptosis induction by the Therien and vaccine RA27/3 strains of rubella virus causes depletion of oligodendrocytes from rat neural cell cultures. J Gen Virol 83:2135–2143. doi:10.1099/0022-1317-83-9-2135 12185266

[84] Lazar M, Perelygina L, Martines R, Greer P, Paddock CD, Peltecu G, Lupulescu E, Icenogle J, Zaki SR. 2016. Immunolocalization and distribution of rubella antigen in fatal congenital rubella syndrome. EBioMedicine 3:86–92. doi:10.1016/j.ebiom.2015.11.050 26870820 PMC4739417

[85] Feng M, Guo S, Fan S, Zeng X, Zhang Y, Liao Y, Wang J, Zhao T, Wang L, Che Y, Wang J, Ma N, Liu L, Yue L, Li Q. 2016. The preferential infection of astrocytes by enterovirus 71 plays a key role in the viral neurogenic pathogenesis. Front Cell Infect Microbiol 6:192. doi:10.3389/fcimb.2016.00192 28066727 PMC5174126

[86] Feng M, Liao Y, Gao Y, Jiang G, Wang L, Zhang Y, Fan S, Xu X, Li Q. 2020. Mechanism for the lethal effect of enterovirus A71 intracerebral injection in neonatal mice. Lab Invest 100:596–605. doi:10.1038/s41374-019-0351-5 31857694 PMC7096333

[87] Zhu P, Ji W, Li D, Wang F, Sun T, Yang H, Chen S, Zhang W, Jin Y, Duan G. 2025. The activation of complement C5a-C5aR1 axis in astrocytes facilitates the neuropathogenesis due to EV-A71 infection by upregulating CXCL1. J Virol 99:e0151424. doi:10.1128/jvi.01514-24 39679722 PMC11784463

[88] Chooi WH, Zeng Y, Lee C-P, Lim ZQ, Gautam P, Chu JJH, Loh Y-H, Alonso S. 2024. Enterovirus-A71 preferentially infects and replicates in human motor neurons, inducing neurodegeneration by ferroptosis. Emerg Microbes Infect 13:2382235. doi:10.1080/22221751.2024.2382235 39017655 PMC11285248

[89] Zou Z, Tsang J-L, Yan B, Chik K-H, Chan C-Y, Cao J, Liang R, Tang K, Yin F, Ye Z-W, Chu H, Chan J-W, Yuan S, Yuen K-Y. 2020. Metabolic profiling reveals significant perturbations of intracellular glucose homeostasis in enterovirus-infected cells. Metabolites 10:302. doi:10.3390/metabo10080302 32717953 PMC7466099

[90] Dyda A, Stelzer-Braid S, Adam D, Chughtai AA, MacIntyre CR. 2018. The association between acute flaccid myelitis (AFM) and Enterovirus D68 (EV-D68) - what is the evidence for causation? Euro Surveill 23:17-00310. doi:10.2807/1560-7917.ES.2018.23.3.17-00310 PMC5792700 29386095

[91] Vogt MR, Wright PF, Hickey WF, De Buysscher T, Boyd KL, Crowe JE. 2022. Enterovirus D68 in the anterior horn cells of a child with acute flaccid myelitis. N Engl J Med 386:2059–2060. doi:10.1056/NEJMc2118155 35613028 PMC9321432

[92] Rosenfeld AB, Warren AL, Racaniello VR. 2019. Neurotropism of Enterovirus D68 isolates is independent of sialic acid and is not a recently acquired phenotype. mBio 10:02370–19. doi:10.1128/mBio.02370-19 PMC6805996 31641090

[93] Liu X, Li H, Li Z, Gao D, Zhou J, Ni F, Yu Q, Huang Y, Tang Y, Xue L, Wang S, Yang J, Guo H, Wang Y, Yu X-F, Yu Z, Wei W. 2025. MFSD6 is an entry receptor for respiratory enterovirus D68. Cell Host & Microbe 33:267–278. doi:10.1016/j.chom.2024.12.015 39798568

[94] Dábilla N, Maya S, McNinch C, Eddens T, Dolan PT, Freeman MC. 2025. Strain-specific tropism and transcriptional responses of enterovirus D68 infection in human spinal cord organoids. Front Microbiol 16:1698639. doi:10.3389/fmicb.2025.1698639 41347238 PMC12672529

[95] Couderc T, Guivel-Benhassine F, Calaora V, Gosselin A-S, Blondel B. 2002. An ex vivo murine model to study poliovirus-induced apoptosis in nerve cells. J Gen Virol 83:1925–1930. doi:10.1099/0022-1317-83-8-1925 12124456

[96] Savagner J, Trémeaux P, Baudou E, Mansuy JM, Cheuret E. 2024. Neurological involvement related to the influenza virus in children: a 5-year single-centre retrospective study. Eur J Paediatr Neurol 51:100–109. doi:10.1016/j.ejpn.2024.05.012 38908343

[97] Davis LE, Koster F, Cawthon A. 2014. Neurologic aspects of influenza viruses. Handb Clin Neurol 123:619–645. doi:10.1016/B978-0-444-53488-0.00030-4 25015508 PMC7152431

[98] Wang G, Zhang J, Li W, Xin G, Su Y, Gao Y, Zhang H, Lin G, Jiao X, Li K. 2008. Apoptosis and proinflammatory cytokine responses of primary mouse microglia and astrocytes induced by human H1N1 and Avian H5N1 influenza viruses. Cell Mol Immunol 5:113–120. doi:10.1038/cmi.2008.14 18445341 PMC4651245

[99] Ng YP, Lee SMY, Cheung TKW, Nicholls JM, Peiris JSM, Ip NY. 2010. Avian influenza H5N1 virus induces cytopathy and proinflammatory cytokine responses in human astrocytic and neuronal cell lines. Neuroscience 168:613–623. doi:10.1016/j.neuroscience.2010.04.013 20398740

[100] Ng YP, Yip TF, Peiris JSM, Ip NY, Lee SMY. 2018. Avian influenza A H7N9 virus infects human astrocytes and neuronal cells and induces inflammatory immune responses. J Neurovirol 24:752–760. doi:10.1007/s13365-018-0659-8 29987581 PMC7094989

[101] Pringproa K, Srivorakul S, Tantilertcharoen R, Thanawongnuwech R. 2018. Restricted infection and cytokine expression in primary murine astrocytes induced by the H5N1 influenza virus. Neurochem J 12:88–94. doi:10.1134/S1819712418010129

[102] Ding X-M, Wang Y-F, Lyu Y, Zou Y, Wang X, Ruan S-M, Wu W-H, Liu H, Sun Y, Zhang R-L, Zhao H, Han Y, Zhao B-T, Pan J, Han X-Y, Wang C-R, Zhao H-L, Yang G-L, Liu L-Z, Fang S-S. 2022. The effect of influenza A (H1N1) pdm09 virus infection on cytokine production and gene expression in BV2 microglial cells. Virus Res 312:198716. doi:10.1016/j.virusres.2022.198716 35240224

[103] Allen IV, McQuaid S, McMahon J, Kirk J, McConnell R. 1996. The significance of measles virus antigen and genome distribution in the CNS in SSPE for mechanisms of viral spread and demyelination. J Neuropathol Exp Neurol 55:471–480. doi:10.1097/00005072-199604000-00010 8786407

[104] Mesquita R, Castaños-Velez E, Biberfeld P, Troian RM, de Siqueira MM. 1998. Measles virus antigen in macrophage/microglial cells and astrocytes of subacute sclerosing panencephalitis. APMIS 106:553–561. doi:10.1111/j.1699-0463.1998.tb01384.x 9674893

[105] Poleshchuk NN, Kvacheva ZB, Ul’kevich YG, Cherstvoi ED. 1996. Infection of highly differentiated human and guinea pig astrocytes with herpes simplex and measles viruses. Bull Exp Biol Med 122:1010–1014. doi:10.1007/BF02447023 9081440

[106] Duprex WP, McQuaid S, Rima BK. 2000. Measles virus-induced disruption of the glial-fibrillary-acidic protein cytoskeleton in an astrocytoma cell line (U-251). J Virol 74:3874–3880. doi:10.1128/JVI.74.8.3874-3880.2000 10729162 PMC111896

[107] Plumb J, Duprex WP, Cameron CHS, Richter-Landsberg C, Talbot P, McQuaid S. 2002. Infection of human oligodendroglioma cells by a recombinant measles virus expressing enhanced green fluorescent protein. J Neurovirol 8:24–34. doi:10.1080/135502802317247785 11847589 PMC7095342

[108] Russell RA, Chojnacki J, Jones DM, Johnson E, Do T, Eggeling C, Padilla-Parra S, Sattentau QJ. 2017. Astrocytes resist HIV-1 fusion but engulf infected macrophage material. Cell Rep 18:1473–1483. doi:10.1016/j.celrep.2017.01.027 28178524 PMC5316642

[109] dos Reis RS, Susa S, Wagner MCE, Ayyavoo V. 2024. Human immunodeficiency virus (HIV-1) targets astrocytes via cell-free and cell-associated infection. J Integr Neurosci 23. doi:10.31083/j.jin2309172 39344243

[110] Wiley CA, Schrier RD, Nelson JA, Lampert PW, Oldstone MB. 1986. Cellular localization of human immunodeficiency virus infection within the brains of acquired immune deficiency syndrome patients. Proc Natl Acad Sci USA 83:7089–7093. doi:10.1073/pnas.83.18.7089 3018755 PMC386658

[111] Tornatore C, Chandra R, Berger JR, Major EO. 1994. HIV‐1 infection of subcortical astrocytes in the pediatric central nervous system. Neurology 44:481–481. doi:10.1212/WNL.44.3_Part_1.481 8145919

[112] Bagasra O, Lavi E, Bobroski L, Khalili K, Pestaner JP, Tawadros R, Pomerantz RJ. 1996. Cellular reservoirs of HIV-1 in the central nervous system of infected individuals: identification by the combination of in situ polymerase chain reaction and immunohistochemistry. AIDS 10:573–585. doi:10.1097/00002030-199606000-00002 8780811

[113] Eugenin EA, Berman JW. 2007. Gap junctions mediate human immunodeficiency virus-bystander killing in astrocytes. J Neurosci 27:12844–12850. doi:10.1523/JNEUROSCI.4154-07.2007 18032656 PMC2117380

[114] Narasipura SD, Kim S, Al-Harthi L. 2014. Epigenetic regulation of HIV-1 latency in astrocytes. J Virol 88:3031–3038. doi:10.1128/JVI.03333-13 24352441 PMC3958059

[115] Lutgen V, Narasipura SD, Barbian HJ, Richards M, Wallace J, Razmpour R, Buzhdygan T, Ramirez SH, Prevedel L, Eugenin EA, Al-Harthi L. 2020. HIV infects astrocytes in vivo and egresses from the brain to the periphery. PLoS Pathog 16:e1008381. doi:10.1371/journal.ppat.1008381 32525948 PMC7289344

[116] Boreland AJ, Stillitano AC, Lin H-C, Abbo Y, Hart RP, Jiang P, Pang ZP, Rabson AB. 2024. Sustained type I interferon signaling after human immunodeficiency virus type 1 infection of human iPSC derived microglia and cerebral organoids. iScience 27:109628. doi:10.1016/j.isci.2024.109628 38628961 PMC11019286

[117] Kong W, Frouard J, Xie G, Corley MJ, Helmy E, Zhang G, Schwarzer R, Montano M, Sohn P, Roan NR, Ndhlovu LC, Gan L, Greene WC. 2024. Neuroinflammation generated by HIV-infected microglia promotes dysfunction and death of neurons in human brain organoids. PNAS Nexus 3:pgae179. doi:10.1093/pnasnexus/pgae179 38737767 PMC11086946

[118] Cosenza MA, Zhao M-L, Si Q, Lee SC. 2002. Human brain parenchymal microglia express CD14 and CD45 and are productively infected by HIV-1 in HIV-1 encephalitis. Brain Pathol 12:442–455. doi:10.1111/j.1750-3639.2002.tb00461.x 12408230 PMC8095974

[119] Castellano P, Prevedel L, Eugenin EA. 2017. HIV-infected macrophages and microglia that survive acute infection become viral reservoirs by a mechanism involving Bim. Sci Rep 7:12866. doi:10.1038/s41598-017-12758-w 28993666 PMC5634422

[120] Tang Y, Chaillon A, Gianella S, Wong LM, Li D, Simermeyer TL, Porrachia M, Ignacio C, Woodworth B, Zhong D, et al.. 2023. Brain microglia serve as a persistent HIV reservoir despite durable antiretroviral therapy. J Clin Invest 133:e167417. doi:10.1172/JCI167417 37317962 PMC10266791

[121] Albright AV, Strizki J, Harouse JM, Lavi E, O’Connor M, González-Scarano F. 1996. HIV-1 infection of cultured human adult oligodendrocytes. Virology 217:211–219. doi:10.1006/viro.1996.0108 8599205

[122] An SF, Groves M, Giometto B, Beckett AAJ, Scaravilli F. 1999. Detection and localisation of HIV-1 DNA and RNA in fixed adult AIDS brain by polymerase chain reaction/in situ hybridisation technique. Acta Neuropathol 98:481–487. doi:10.1007/s004010051113 10541871

[123] Schwartz L, Civitello L, Dunn-Pirio A, Ryschkewitsch S, Berry E, Cavert W, Kinzel N, Lawrence DMP, Hazra R, Major EO. 2007. Evidence of human immunodeficiency virus type 1 infection of nestin-positive neural progenitors in archival pediatric brain tissue. J Neurovirol 13:274–283. doi:10.1080/13550280701344975 17613718

[124] Balinang JM, Masvekar RR, Hauser KF, Knapp PE. 2017. Productive infection of human neural progenitor cells by R5 tropic HIV-1: opiate co-exposure heightens infectivity and functional vulnerability. AIDS 31:753–764. doi:10.1097/QAD.0000000000001398 28099189 PMC5342937

[125] Lawrence DMP, Durham LC, Schwartz L, Seth P, Maric D, Major EO. 2004. Human Immunodeficiency virus type 1 infection of human brain-derived progenitor cells. J Virol 78:7319–7328. doi:10.1128/JVI.78.14.7319-7328.2004 15220405 PMC434111

[126] Menet A, Speth C, Larcher C, Prodinger WM, Schwendinger MG, Chan P, Jäger M, Schwarzmann F, Recheis H, Fontaine M, Dierich MP. 1999. Epstein-Barr virus infection of human astrocyte cell lines. J Virol 73:7722–7733. doi:10.1128/JVI.73.9.7722-7733.1999 10438862 PMC104299

[127] Rashidi AS, Tran DN, Peelen CR, van Gent M, Ouwendijk WJD, Verjans GMGM. 2024. Herpes simplex virus infection induces necroptosis of neurons and astrocytes in human fetal organotypic brain slice cultures. J Neuroinflammation 21:38. doi:10.1186/s12974-024-03027-5 38302975 PMC10832279

[128] Słońska A, Cymerys J, Chodkowski M, Bąska P, Krzyżowska M, Bańbura MW. 2021. Human herpesvirus type 2 infection of primary murine astrocytes causes disruption of the mitochondrial network and remodeling of the actin cytoskeleton: an in vitro morphological study. Arch Virol 166:1371–1383. doi:10.1007/s00705-021-05025-x 33715038 PMC8036217

[129] Bansode YD, Chattopadhyay D, Saha B. 2019. Innate immune response in astrocytes infected with herpes simplex virus 1. Arch Virol 164:1433–1439. doi:10.1007/s00705-019-04197-x 30868265

[130] Michael BD, Bricio-Moreno L, Sorensen EW, Miyabe Y, Lian J, Solomon T, Kurt-Jones EA, Luster AD. 2020. Astrocyte- and neuron-derived CXCL1 drives neutrophil transmigration and blood-brain barrier permeability in viral encephalitis. Cell Rep 32:108150. doi:10.1016/j.celrep.2020.108150 32937134 PMC7548103

[131] Bello-Morales R, Crespillo AJ, García B, Dorado LÁ, Martín B, Tabarés E, Krummenacher C, de Castro F, López-Guerrero JA. 2014. The effect of cellular differentiation on HSV-1 infection of oligodendrocytic cells. PLoS One 9:e89141. doi:10.1371/journal.pone.0089141 24551233 PMC3923881

[132] Kastrukoff LF, Kim SU. 2002. Oligodendrocytes from human donors differ in resistance to herpes simplex virus 1 (HSV-1). Glia 38:87–92. doi:10.1002/glia.10043 11921206

[133] López-Guerrero JA, de la Nuez C, Praena B, Sánchez-León E, Krummenacher C, Bello-Morales R. 2020. Herpes simplex virus 1 spread in oligodendrocytic cells is highly dependent on MAL proteolipid. J Virol 94:e01739-19. doi:10.1128/JVI.01739-19 31748392 PMC6997773

[134] Bello-Morales R, Praena B, de la Nuez C, Rejas MT, Guerra M, Galán-Ganga M, Izquierdo M, Calvo V, Krummenacher C, López-Guerrero JA. 2018. Role of microvesicles in the spread of herpes simplex virus 1 in oligodendrocytic cells. J Virol 92:00088–18. doi:10.1128/JVI.00088-18 PMC5923088 29514899

[135] Zheng W, Klammer AM, Naciri JN, Yeung J, Demers M, Milosevic J, Kinchington PR, Bloom DC, Nimgaonkar VL, D’Aiuto L. 2020. Patterns of herpes simplex virus 1 infection in neural progenitor cells. J Virol 94:e00994-20. doi:10.1128/JVI.00994-20 32493817 PMC7394888

[136] Salgado B, Sastre I, Bullido MJ, Aldudo J. 2023. Herpes simplex virus type 1 induces AD-like neurodegeneration markers in human progenitor and differentiated ReNcell VM Cells. Microorganisms 11:1205. doi:10.3390/microorganisms11051205 37317179 PMC10221020

[137] Wood JA, Chaparala S, Bantang C, Chattopadhyay A, Wesesky MA, Kinchington PR, Nimgaonkar VL, Bloom DC, D’Aiuto L. 2024. RNA-Seq time-course analysis of neural precursor cell transcriptome in response to herpes simplex Virus-1 infection. J Neurovirol 30:131–145. doi:10.1007/s13365-024-01198-8 38478163 PMC11371869

[138] Lecointe D, Héry C, Janabi N, Dussaix E, Tardieu M. 1999. Differences in kinetics of human cytomegalovirus cell-free viral release after in vitro infection of human microglial cells, astrocytes and monocyte-derived macrophages. J Neurovirol 5:308–313. doi:10.3109/13550289909015817 10414521

[139] Lokensgard JR, Cheeran MC, Gekker G. 1999. Human cytomegalovirus replication and modulation of apoptosis in astrocytes. J Hum Virol 2:91–101. 10225211

[140] Maxim C-J C, Shuxian H. 2001. Cytomegalovirus induces cytokine and chemokine production differentially in microglia and astrocytes: antiviral implications. J Neurovirol 7:135–147. doi:10.1080/13550280152058799 11517386

[141] Pulliam L. 1991. Cytomegalovirus preferentially infects a monocyte derived macrophage/microglial cell in human brain cultures. J Neuropathol Exp Neurol 50:432–440. doi:10.1097/00005072-199107000-00004 1648122

[142] Teissier N, Fallet-Bianco C, Delezoide A-L, Laquerrière A, Marcorelles P, Khung-Savatovsky S, Nardelli J, Cipriani S, Csaba Z, Picone O, Golden JA, Van Den Abbeele T, Gressens P, Adle-Biassette H. 2014. Cytomegalovirus-Induced Brain Malformations in Fetuses. Journal of Neuropathology & Experimental Neurology 73:143–158. doi:10.1097/NEN.0000000000000038 24423639

[143] Pan X, Li X-J, Liu X-J, Yuan H, Li J-F, Duan Y-L, Ye H-Q, Fu Y-R, Qiao G-H, Wu C-C, Yang B, Tian X-H, Hu K-H, Miao L-F, Chen X-L, Zheng J, Rayner S, Schwartz PH, Britt WJ, Xu J, Luo M-H. 2013. Later passages of neural progenitor cells from neonatal brain are more permissive for human cytomegalovirus infection. J Virol 87:10968–10979. doi:10.1128/JVI.01120-13 23903847 PMC3807278

[144] Luo MH, Hannemann H, Kulkarni AS, Schwartz PH, O’Dowd JM, Fortunato EA. 2010. Human cytomegalovirus infection causes premature and abnormal differentiation of human neural progenitor cells. J Virol 84:3528–3541. doi:10.1128/JVI.02161-09 20071566 PMC2838134

[145] Wu C-C, Jiang X, Wang X-Z, Liu X-J, Li X-J, Yang B, Ye H-Q, Harwardt T, Jiang M, Xia H-M, Wang W, Britt WJ, Paulus C, Nevels M, Luo M-H. 2018. Human cytomegalovirus immediate early 1 protein causes loss of SOX2 from neural progenitor cells by trapping unphosphorylated STAT3 in the nucleus. J Virol 92:00340–18. doi:10.1128/JVI.00340-18 PMC6096794 29950413

[146] Jiang X, Liu S, Fu Y-R, Liu X-J, Li X-J, Yang B, Jiang H-F, Shen Z-Z, Alemu EA, Vazquez P, Tang Y, Kaarbø M, McVoy MA, Rayner S, Luo M-H. 2023. Human cytomegalovirus infection perturbs neural progenitor cell fate via the expression of viral microRNAs. J Med Virol 95:e28574. doi:10.1002/jmv.28574 36772841 PMC12812264

[147] Odeberg J, Wolmer N, Falci S, Westgren M, Seiger A, Söderberg-Nauclér C. 2006. Human cytomegalovirus inhibits neuronal differentiation and induces apoptosis in human neural precursor cells. J Virol 80:8929–8939. doi:10.1128/JVI.00676-06 16940505 PMC1563895

[148] Assouline JG, Levin MJ, Major EO, Forghani B, Straus SE, Ostrove JM. 1990. Varicella-zoster virus infection of human astrocytes, Schwann cells, and neurons. Virology 179:834–844. doi:10.1016/0042-6822(90)90152-h 2173263

[149] Bubak AN, Como CN, Blackmon AM, Jones D, Nagel MA. 2018. Varicella zoster virus differentially alters morphology and suppresses proinflammatory cytokines in primary human spinal cord and hippocampal astrocytes. J Neuroinflammation 15:318. doi:10.1186/s12974-018-1360-9 30442152 PMC6236967

[150] Moulignier A, Pialoux G, Dega H, Dupont B, Huerre M, Baudrimont M. 1995. Brain stem encephalitis due to varicella-zoster virus in a patient with AIDS. Clin Infect Dis 20:1378–1380. doi:10.1093/clinids/20.5.1378 7620026

[151] Carpenter JE, Clayton AC, Halling KC, Bonthius DJ, Buckingham EM, Jackson W, Dotzler SM, Card JP, Enquist LW, Grose C. 2016. Defensive perimeter in the central nervous system: predominance of astrocytes and astrogliosis during recovery from varicella-zoster virus encephalitis. J Virol 90:379–391. doi:10.1128/JVI.02389-15 26491149 PMC4702565

[152] Devinsky O, Cho ES, Petito CK, Price RW. 1991. Herpes zoster myelitis. Brain 114 ( Pt 3):1181–1196. doi:10.1093/brain/114.3.1181 1648419

[153] Barah F, Whiteside S, Batista S, Morris J. 2014. Neurological aspects of human parvovirus B19 infection: a systematic review. Rev Med Virol 24:154–168. doi:10.1002/rmv.1782 24459081 PMC4238837

[154] Skuja S, Vilmane A, Svirskis S, Groma V, Murovska M. 2018. Evidence of human parvovirus B19 infection in the post-mortem brain tissue of the elderly. Viruses 10:582. doi:10.3390/v10110582 30366357 PMC6267580

[155] Isumi H, Nunoue T, Nishida A, Takashima S. 1999. Fetal brain infection with human parvovirus B19. Pediatr Neurol 21:661–663. doi:10.1016/s0887-8994(99)00055-7 10513695

[156] Major EO, Vacante DA. 1989. Human fetal astrocytes in culture support the growth of the neurotropic human polyomavirus, JCV. J Neuropathol Exp Neurol 48:425–436. doi:10.1097/00005072-198907000-00004 2543798

[157] Kondo Y, Windrem MS, Zou L, Chandler-Militello D, Schanz SJ, Auvergne RM, Betstadt SJ, Harrington AR, Johnson M, Kazarov A, Gorelik L, Goldman SA. 2014. Human glial chimeric mice reveal astrocytic dependence of JC virus infection. J Clin Invest 124:5323–5336. doi:10.1172/JCI76629 25401469 PMC4348956

[158] Honkimaa A, Laine P, Suppula J, Tynninen O, Saarela M, Laakso SM, Hetemäki I, Liimatainen H, Auvinen P, Auvinen E. 2024. Exploring JC polyomavirus sequences and human gene expression in brain tissue of patients with progressive multifocal leukoencephalopathy. J Infect Dis 230:e732–e736. doi:10.1093/infdis/jiae066 38365889 PMC11420775

[159] Radhakrishnan S, Otte J, Enam S, Del Valle L, Khalili K, Gordon J. 2003. JC virus-induced changes in cellular gene expression in primary human astrocytes. J Virol 77:10638–10644. doi:10.1128/JVI.77.19.10638-10644.2003 12970448 PMC228539

[160] Oberholster L, Mathias A, Perriot S, Blaser E, Canales M, Jones S, Culebras L, Gimenez M, Kaynor GC, Sapozhnik A, Richetin K, Goelz S, Du Pasquier R. 2023. Comprehensive proteomic analysis of JC polyomavirus-infected human astrocytes and their extracellular vesicles. Microbiol Spectr 11:e0275123. doi:10.1128/spectrum.02751-23 37815349 PMC10714778

[161] Wilczek MP, Armstrong FJ, Geohegan RP, Mayberry CL, DuShane JK, King BL, Maginnis MS. 2021. The MAPK/ERK pathway and the role of DUSP1 in JCPyV infection of primary astrocytes. Viruses 13:1834. doi:10.3390/v13091834 34578413 PMC8473072

[162] Peterson JN, Lin B, Shin J, Phelan PJ, Tsichlis P, Schwob JE, Bullock PA. 2017. Replication of JC virus DNA in the G144 oligodendrocyte cell line is dependent upon Akt. J Virol 91:00735–17. doi:10.1128/JVI.00735-17 PMC5625518 28768870

[163] Richardson-Burns SM, Kleinschmidt-DeMasters BK, DeBiasi RL, Tyler KL. 2002. Progressive multifocal leukoencephalopathy and apoptosis of infected oligodendrocytes in the central nervous system of patients with and without AIDS. Arch Neurol 59:1930. doi:10.1001/archneur.59.12.1930 12470182

[164] Merabova N, Kaniowska D, Kaminski R, Deshmane SL, White MK, Amini S, Darbinyan A, Khalili K. 2008. JC virus agnoprotein inhibits in vitro differentiation of oligodendrocytes and promotes apoptosis. J Virol 82:1558–1569. doi:10.1128/JVI.01680-07 17989177 PMC2224429

[165] Piña-Oviedo S, Urbanska K, Radhakrishnan S, Sweet T, Reiss K, Khalili K, Del Valle L. 2007. Effects of JC virus infection on anti-apoptotic protein survivin in progressive multifocal leukoencephalopathy. Am J Pathol 170:1291–1304. doi:10.2353/ajpath.2007.060689 17392168 PMC1829462

[166] Del Valle L, Sweet T, Parker-Struckhoff A, Perez-Liz G, Piña-Oviedo S. 2020. JCPyV T-antigen activation of the anti-apoptotic survivin promoter-its role in the development of progressive multifocal leukoencephalopathy. Viruses 12:1253. doi:10.3390/v12111253 33153187 PMC7693140

[167] Darbinyan A, Kaminski R, White MK, Darbinian-Sarkissian N, Khalili K. 2013. Polyomavirus JC infection inhibits differentiation of oligodendrocyte progenitor cells. J Neurosci Res 91:116–127. doi:10.1002/jnr.23135 23086711 PMC4641310

[168] Li C, Huynh NPT, Schanz SJ, Windrem MS, Goldman SA. 2024. JC virus spread is potentiated by glial replication and demyelination-linked glial proliferation. Brain 147:4131–4146. doi:10.1093/brain/awae252 39133566 PMC12098017

[169] Schultz-Pernice I, Fahmi A, Brito F, Liniger M, Chiu Y-C, David T, Oliveira Esteves BI, Golomingi A, Zumkehr B, Gerber M, Jandrasits D, Züst R, Steiner S, Wotzkow C, Blank F, Engler OB, Summerfield A, Ruggli N, Baud D, Alves MP. 2025. Monkeypox virus spreads from cell-to-cell and leads to neuronal death in human neural organoids. Nat Commun 16:5376. doi:10.1038/s41467-025-61134-0 40588500 PMC12209443

[170] Miranzadeh Mahabadi H, Lin YCJ, Ogando NS, Moussa EW, Mohammadzadeh N, Julien O, Alto NM, Noyce RS, Evans DH, Power C. 2024. Monkeypox virus infection of human astrocytes causes gasdermin B cleavage and pyroptosis. Proc Natl Acad Sci USA 121. doi:10.1073/pnas.2315653121 PMC10895262 38346199

[171] Endo F, Kasai A, Soto JS, Yu X, Qu Z, Hashimoto H, Gradinaru V, Kawaguchi R, Khakh BS. 2022. Molecular basis of astrocyte diversity and morphology across the CNS in health and disease. Science 378:eadc9020. doi:10.1126/science.adc9020 36378959 PMC9873482

[172] Gradisnik L, Velnar T. 2023. Astrocytes in the central nervous system and their functions in health and disease: a review. World J Clin Cases 11:3385–3394. doi:10.12998/wjcc.v11.i15.3385 37383914 PMC10294192

[173] Verkhratsky A, Parpura V, Li B, Scuderi C. 2021. Astrocytes: the housekeepers and guardians of the CNS. Adv Neurobiol 26:21–53. doi:10.1007/978-3-030-77375-5_2 34888829 PMC9004589

[174] Jorgačevski J, Potokar M. 2023. Immune functions of astrocytes in viral neuroinfections. Int J Mol Sci 24:3514. doi:10.3390/ijms24043514 36834929 PMC9960577

[175] Sofroniew MV. 2020. Astrocyte reactivity: subtypes, states, and functions in CNS innate immunity. Trends Immunol 41:758–770. doi:10.1016/j.it.2020.07.004 32819810 PMC7484257

[176] Escartin C, Galea E, Lakatos A, O’Callaghan JP, Petzold GC, Serrano-Pozo A, Steinhäuser C, Volterra A, Carmignoto G, Agarwal A, et al.. 2021. Reactive astrocyte nomenclature, definitions, and future directions. Nat Neurosci 24:312–325. doi:10.1038/s41593-020-00783-4 33589835 PMC8007081

[177] Galland F, Seady M, Taday J, Smaili SS, Gonçalves CA, Leite MC. 2019. Astrocyte culture models: molecular and function characterization of primary culture, immortalized astrocytes and C6 glioma cells. Neurochem Int 131:104538. doi:10.1016/j.neuint.2019.104538 31430518

[178] Preato AM, Pinheiro E da S, Rosenstock TR, Glezer I. 2024. The relevance of astrocytic cell culture models for neuroinflammation in neurodegeneration research. Neuroglia 5:27–49. doi:10.3390/neuroglia5010003

[179] Mulica P, Venegas C, Landoulsi Z, Badanjak K, Delcambre S, Tziortziou M, Hezzaz S, Ghelfi J, Smajic S, Schwamborn J, Krüger R, Antony P, May P, Glaab E, Grünewald A, Pereira SL. 2023. Comparison of two protocols for the generation of iPSC-derived human astrocytes. Biol Proced Online 25:26. doi:10.1186/s12575-023-00218-x 37730545 PMC10512486

[180] Lendemeijer B, Unkel M, Smeenk H, Mossink B, Hijazi S, Gordillo-Sampedro S, Shpak G, Slump DE, van den Hout MCGN, van IJcken WFJ, Bindels EMJ, Hoogendijk WJG, Nadif Kasri N, de Vrij FMS, Kushner SA. 2024. Human pluripotent stem cell-derived astrocyte functionality compares favorably with primary rat astrocytes. eNeuro 11:ENEURO.0148-24.2024. doi:10.1523/ENEURO.0148-24.2024 PMC11404293 39227152

[181] Lange SC, Bak LK, Waagepetersen HS, Schousboe A, Norenberg MD. 2012. Primary cultures of astrocytes: their value in understanding astrocytes in health and disease. Neurochem Res 37:2569–2588. doi:10.1007/s11064-012-0868-0 22926576 PMC4737437

[182] Depla JA, Mulder LA, de Sá RV, Wartel M, Sridhar A, Evers MM, Wolthers KC, Pajkrt D. 2022. Human brain organoids as models for central nervous system viral infection. Viruses 14:634. doi:10.3390/v14030634 35337041 PMC8948955

[183] Marques V de M, Santos CS, Santiago IG, Marques SM, Nunes Brasil M das G, Lima TT, Costa PS. 2019. Neurological complications of congenital zika virus infection. Pediatr Neurol 91:3–10. doi:10.1016/j.pediatrneurol.2018.11.003 30591235

[184] Muñoz LS, Parra B, Pardo CA, NEitA S. 2017. Neurological implications of zika virus infection in adults. J Infect Dis 216:S897–S905. doi:10.1093/infdis/jix511 29267923 PMC5853915

[185] Hygino da Cruz LC Jr, Nascimento OJM, Lopes FPPL, da Silva IRF. 2018. Neuroimaging findings of zika virus–associated neurologic complications in adults. AJNR Am J Neuroradiol 39:1967–1974. doi:10.3174/ajnr.A5649 29773562 PMC7655374

[186] Mlakar J, Korva M, Tul N, Popović M, Poljšak-Prijatelj M, Mraz J, Kolenc M, Resman Rus K, Vesnaver Vipotnik T, Fabjan Vodušek V, Vizjak A, Pižem J, Petrovec M, Avšič Županc T. 2016. Zika virus associated with microcephaly. N Engl J Med 374:951–958. doi:10.1056/NEJMoa1600651 26862926

[187] Scotto G, Massa S, Spirito F, Fazio V. 2023. Congenital zika virus syndrome: microcephaly and orofacial anomalies. Life (Basel) 14:55. doi:10.3390/life14010055 38255670 PMC10820182

[188] Megli CJ, Coyne CB. 2022. Infections at the maternal-fetal interface: an overview of pathogenesis and defence. Nat Rev Microbiol 20:67–82. doi:10.1038/s41579-021-00610-y 34433930 PMC8386341

[189] Palus M, Bílý T, Elsterová J, Langhansová H, Salát J, Vancová M, Růžek D. 2014. Infection and injury of human astrocytes by tick-borne encephalitis virus. J Gen Virol 95:2411–2426. doi:10.1099/vir.0.068411-0 25000960

[190] Freeman MC, Messacar K. 2025. Enterovirus and parechovirus neurologic infections in children: clinical presentations and neuropathogenesis. J Pediatric Infect Dis Soc 14:piae069. doi:10.1093/jpids/piae069 39776161

[191] Ferren M, Horvat B, Mathieu C. 2019. Measles encephalitis: towards new therapeutics. Viruses 11:1017. doi:10.3390/v11111017 31684034 PMC6893791

[192] Gelman BB. 2015. Neuropathology of HAND with suppressive antiretroviral therapy: encephalitis and neurodegeneration reconsidered. Curr HIV/AIDS Rep 12:272–279. doi:10.1007/s11904-015-0266-8 25860316 PMC4427627

[193] Levintov L, Vashisth H. 2024. Structural and computational studies of HIV-1 RNA. RNA Biol 21:1–32. doi:10.1080/15476286.2023.2289709 PMC10730233 38100535

[194] Carneiro VC de S, Pereira JG, de Paula VS. 2022. Family Herpesviridae and neuroinfections: current status and research in progress. Mem Inst Oswaldo Cruz 117:e220200. doi:10.1590/0074-02760220200 36417627 PMC9677594

[195] Xia W, Yan H, Zhang Y, Wang C, Gao W, Lv C, Wang W, Liu Z. 2021. Congenital human cytomegalovirus infection inducing sensorineural hearing loss. Front Microbiol 12:2021. doi:10.3389/fmicb.2021.649690 PMC8079719 33936007

[196] Bhandari G, Acharya A, Chettri AK, Sharma S. 2025. Neurological manifestations of Mpox virus during the recent global outbreak: a systematic review. BMC Infect Dis 25:1188. doi:10.1186/s12879-025-11631-w 41023645 PMC12482380

[197] Butic AB, Spencer SA, Shaheen SK, Lukacher AE. 2023. Polyomavirus wakes up and chooses neurovirulence. Viruses 15:2112. doi:10.3390/v15102112 37896889 PMC10612099

[198] Colonna M, Butovsky O. 2017. Microglia function in the central nervous system during health and neurodegeneration. Annu Rev Immunol 35:441–468. doi:10.1146/annurev-immunol-051116-052358 28226226 PMC8167938

[199] Ismail FS, Faustmann TJ, Faustmann PM, Corvace F. 2024. Microglia as potential key regulators in viral-induced neuroinflammation. Front Cell Neurosci 18:1426079. doi:10.3389/fncel.2024.1426079 39055547 PMC11269195

[200] Woolf Z, Stevenson TJ, Lee K, Highet B, Macapagal Foliaki J, Ratiu R, Rustenhoven J, Correia J, Schweder P, Heppner P, Weinert M, Coppieters N, Park T, Montgomery J, Smith AM, Dragunow M. 2025. In vitro models of microglia: a comparative study. Sci Rep 15:15621. doi:10.1038/s41598-025-99867-z 40320508 PMC12050316

[201] McMillan RE, Wang E, Carlin AF, Coufal NG. 2023. Human microglial models to study host–virus interactions. Exp Neurol 363:114375. doi:10.1016/j.expneurol.2023.114375 36907350 PMC10521930

[202] Di Stefano J, Di Marco F, Cicalini I, FitzGerald U, Pieragostino D, Verhoye M, Ponsaerts P, Van Breedam E. 2025. Generation, interrogation, and future applications of microglia-containing brain organoids. Neural Regen Res 20:3448–3460. doi:10.4103/NRR.NRR-D-24-00921 39665813 PMC11974650

[203] Zhang N, Yi R, Zhong F, Lu Y, Chen W, Ke Z, Zhang Y, Zhou L, Wang P, Li W. 2025. Oligodendrocytes and myelination: pioneering new frontiers in cognitive neuroscience. Front Neurosci 19:2025. doi:10.3389/fnins.2025.1618468 PMC12319009 40761314

[204] Roussarie J-P, Ruffié C, Brahic M. 2007. The role of myelin in theiler’s virus persistence in the central nervous system. PLoS Pathog 3:e23. doi:10.1371/journal.ppat.0030023 17305428 PMC1797621

[205] Pan R, Zhang Q, Anthony SM, Zhou Y, Zou X, Cassell M, Perlman S. 2020. Oligodendrocytes that survive acute coronavirus infection induce prolonged inflammatory responses in the CNS. Proc Natl Acad Sci USA 117:15902–15910. doi:10.1073/pnas.2003432117 32571951 PMC7355048

[206] De Kleijn KMA, Zuure WA, Peijnenborg J, Heuvelmans JM, Martens GJM. 2019. Reappraisal of human HOG and MO3.13 cell lines as a model to study oligodendrocyte functioning. Cells 8:1096. doi:10.3390/cells8091096 31533280 PMC6769895

[207] Ladran I, Tran N, Topol A, Brennand KJ. 2013. Neural stem and progenitor cells in health and disease. WIREs Mechanisms of Disease 5:701–715. doi:10.1002/wsbm.1239 PMC4160040 24068527

[208] Miller LN, Walters AE, Denninger JK, Hanson MA, Marshall AH, Johantges AC, Hosawi M, Sebring G, Rieskamp JD, Ding T, Rindani R, Chen KS, Goldberg ME, Senthilvelan S, Volk A, Zhao F, Askwith C, Wester JC, Kirby ED. 2025. Neural stem and progenitor cells support and protect adult hippocampal function via vascular endothelial growth factor secretion. Mol Psychiatry 30:2152–2167. doi:10.1038/s41380-024-02827-8 39528687 PMC12014380

[209] Behnan J, Stangeland B, Langella T, Finocchiaro G, Tringali G, Meling TR, Murrell W. 2017. Identification and characterization of a new source of adult human neural progenitors. Cell Death Dis 8:e2991–e2991. doi:10.1038/cddis.2017.368 28796246 PMC5596556

[210] Donato R, Miljan EA, Hines SJ, Aouabdi S, Pollock K, Patel S, Edwards FA, Sinden JD. 2007. Differential development of neuronal physiological responsiveness in two human neural stem cell lines. BMC Neurosci 8:36. doi:10.1186/1471-2202-8-36 17531091 PMC1888696

[211] Howard-Jones AR, Pham D, Sparks R, Maddocks S, Dwyer DE, Kok J, Basile K. 2023. Arthropod-borne flaviviruses in pregnancy. Microorganisms 11:433. doi:10.3390/microorganisms11020433 36838398 PMC9959669

